# Social Housing Leads to Increased Ethanol Intake in Male Mice Housed in Environmentally Enriched Cages

**DOI:** 10.3389/fnbeh.2021.695409

**Published:** 2021-06-18

**Authors:** Hannah D. Fulenwider, Meridith T. Robins, Maya A. Caruso, Andrey E. Ryabinin

**Affiliations:** Department of Behavioral Neuroscience, Oregon Health and Science University, Portland, OR, United States

**Keywords:** radiofrequency identification, RFID, C57BL/6J mice, HM2, corticotropin releasing hormone, FosB

## Abstract

An individual's social environment affects alcohol intake. However, the complex interactions between social context and alcohol intake remain understudied in preclinical models. In the present study, we sought to characterize the effects of social housing on voluntary ethanol intake in male C567BL/6J mice using a continuous access two-bottle choice model. This was accomplished using HM2 cages, which allow for the continuous monitoring of individuals' fluid intake through radiofrequency tracking while they remain undisturbed in a group setting. These cages are moderately environmentally enriched compared to standard shoebox cages. By analyzing the levels of voluntary ethanol intake between socially- and individually-housed mice in HM2 cages, we were able to parse apart the effects of environmental enrichment vs. social enrichment. We found that while intake levels were overall lower than those observed when animals are singly housed in standard shoebox cages, socially-housed males consumed significantly more ethanol compared to individually-housed mice, suggesting that while environmental enrichment attenuates ethanol intake, social enrichment may, in fact, potentiate it. This effect was not specific for alcohol, however, in that ethanol preference did not differ as a product of social context. We also found that the total number of non-consummatory channel entries were consistently higher in individually-housed mice. Additionally, a single corticotropin releasing factor receptor 1 antagonist treatment significantly decreased both water and ethanol intake in socially- and individually-housed mice up to 3 h post-treatment, though the effect on water intake was longer lasting. This treatment also significantly decreased the number of non-consummatory channel entries in individually-housed mice, but not in socially-housed mice, suggesting that increased channel visits may be a stress-related behavior. Lastly, we examined blood ethanol concentrations and FosB immunoreactivity to characterize the physiological responses to ethanol intake in socially- and individually-housed mice. The number of FosB-positive cells in the centrally-projecting Edinger-Westphal nucleus and nucleus accumbens shell positively correlated with average baseline ethanol intake in individually-housed mice, but not in socially-housed mice. Overall, we found that social, but not environmental, enrichment can increase ethanol intake in male C57BL/6J mice. Future studies need to test this phenomenon in female mice and assess the generalizability of this finding.

## Introduction

Social environment can have profound effects on alcohol drinking. On one hand, peer pressure can lead to excessive alcohol consumption. On the other hand, social support is frequently used to decrease problematic drinking. Understanding mechanisms underlying the effects of social environment on alcohol drinking can aid in the development of new strategies to treat alcohol use disorder (AUD). Animal models could be invaluable for deciphering such mechanisms. However, the majority of preclinical alcohol studies use individually-housed rodents to examine mechanisms of AUD or its therapeutic treatments. This caveat is problematic as recent animal research clearly indicates that social reward can modulate or even interfere with drug-related reward (Venniro et al., 2018; Pelloux et al., 2019; El Rawas et al., 2020). Therefore, studies of the mechanisms regulating alcohol consumption in social settings are greatly needed.

Unfortunately, accurately monitoring ethanol drinking in social settings is a difficult task (Ryabinin and Walcott, 2018). For example, giving socially-housed animals access to ethanol solutions in the standard cage settings prevents the measure of individual levels of intake. Testing animals is semi-social conditions, in which individuals are separated by a semi-translucent permeable barrier, was initially thought to overcome this caveat (Anacker et al., 2011). However, studies showing greater stress-like responses in such conditions than in socially- or individually-housed animals suggest that data from these of studies warrant cautious/conservative interpretation (Van Loo et al., 2007). Additional studies have used video-tracking/scoring, allowing for the analysis of time spent at the ethanol bottle for each animal (Logue et al., 2014). Further, more sophisticated studies began to employ radiofrequency tracking in conjunction with Intellicage systems (TSE Systems), which record the total number of approaches, nose-pokes, and licks at ethanol the ethanol spout for individual animals within a social setting (Radwanska and Kaczmarek, 2012). However, potential spillage in these approaches is not possible to account for, and the fact that ethanol access requires subjects to complete an operant behavior suggests that these studies are more akin to those assessing operant ethanol self-administration rather than voluntary two-bottle choice. Additionally, while a major technological advancement, studies using the Intellicage system have often observed very low ethanol preference for the alcohol spout in socially-housed mice (Holgate et al., 2017; Koskela et al., 2018). This finding suggests that social enrichment may prevent meaningful modeling of ethanol drinking in mice, warranting further investigation.

Recently, a new radiotracking system called Herdsman-2 (HM2) has been developed to allow for more precise monitoring of individual fluid intake. The system was initially developed to track food consumption in preclinical obesity research by matching animal's proximity to the food trough with measurement of weight changes of the provided food using a precision balance (Axel et al., 2010; Thomsen et al., 2017). The system was then modified to measure fluid consumption and adapted to study ethanol consumption (Thomsen et al., 2017; Walcott and Ryabinin, 2020). The system consists of a large communal cage with two protruding channels. Each channel leads to a bottle/ drinking spout and can only be occupied by a single animal at any given time. The bottles are located on an automated precision balance. Photoelements detect the presence of the microchipped animal in the proximity of the spout as the balance measures displacement of fluid. Spillage and evaporation are automatically zeroed out to provide exact measurement of intake. Our lab has recently used this system to assess the effects of repeated oxytocin treatment on voluntary ethanol intake in group-housed male and female mice (Caruso et al., 2021). While a significant, attenuating effect of oxytocin treatment on ethanol intake was observed in this study, it was clear that mice housed socially in the HM2 system exhibited markedly lower levels of intake than those typically observed in mice housed individually in standard shoebox cages. However, a direct assessment of the effects of social/environmental enrichment vs. environmental enrichment alone have yet to be assessed.

To address this question, we exposed individually- and socially-housed male C57BL/6J mice to continuous access ethanol two bottle choice (2BC) in the HM2 system. We also conducted immunohistochemistry for FosB across four brain regions to verify whether ethanol consumption in these cages resulted in changes in neural activity. Lastly, we tested whether one of mechanisms involved in regulation of alcohol intake would be differentially sensitive to pharmacological manipulations in singly- vs. socially-housed animals. Specifically, a substantial number of previous studies suggested that social behaviors are regulated by the corticotropin releasing system reviewed in (Takahashi et al., 2012; Hostetler and Ryabinin, 2013). Therefore, we tested the effects of CP-376,395, a corticotropin releasing factor receptor 1 (CRFR1) antagonist known to decrease ethanol intake in singly-housed mice (Giardino and Ryabinin, 2013; Potretzke et al., 2020).

## Materials and Methods

### Animals

8-week-old C57BL/6J male mice (*n* = 24 per housing condition, *n* = 12 per treatment/housing condition group) were purchased from Jackson Laboratories (Bar Harbor, ME, USA) and allowed one week to habituate to our facilities. Upon arrival, mice were housed in groups of 4-5 in standard “shoebox” cages (18.4 cm W x 29.2 cm D x 12.7 cm H) with food and water provided ab libitum. The colony room was maintained on a standard 12:12 light cycle (lights on 7:00; off 19:00). All experiments were approved by the Oregon Health & Science University animal care and use committee and performed under the guidelines of the National Institute of Health Guidelines for Care and Use of Laboratory animals and the Guidelines for the Care and Use of Mammals in Neuroscience and Behavioral Research.

### RFID Implantation

Directly prior to transfer into the reverse light cycle room, animals were implanted subcutaneously behind the shoulders with RFID chips (UNO Pico ID Transponder (7 mm, UNO-PICO-7), Med Associates, Fairfax VT USA) under light isoflurane anesthesia. Successful RFID implantation was verified using HM2 RFID scanner (MBRose, Faaborg, Denmark) to assure proper placement and function. Following implantation, animals were returned to their homecage and moved into the reverse light cycle suite (lights off 11:00, on 23:00) for up to 48 h of recovery. During this time, food and water were provided ad libitum.

### HM2 Cages

After 2 days of recovery from RFID implantation, mice were moved into HM2 cages (MBRose), measuring 48 x 37.5 x 21 cm and described in detail in (Caruso et al., 2021). Since these cages are larger than standard size, contain shredded paper and cotton nestlet materials, a plastic tube and two built-in fluid access channels, they comprise a moderately enriched environment. Additionally, these cages allow 24 h precise monitoring of fluid consumption in radiotracked mice while avoiding potential confounds of fluid spillage and evaporation. Four mice per cage were housed in each “social” cage, while one mouse per cage was housed in each “individual” cage. The laboratory is equipped with 6 HM2 cages, and we ran our experiments in 5 cohorts. Each cohort included 4-5 cages of individually-housed mice and 1–2 cages of socially-housed mice. A 5-day habituation session was conducted at this time, where both fluid channels contained autoclaved water, and daily consumption was monitored to assure that animals were consuming fluid.

**Figure 1:**
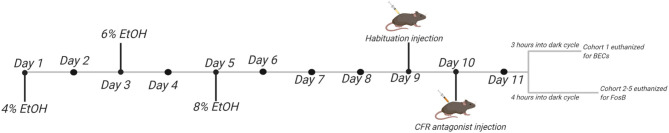
Schematic of experimental timeline.

Following 5 days of habituation to the HM2 cages (and a total of 7 days of habituation to the reverse light cycle), one water channel was randomly assigned to be replaced with 4% ethanol solution (95% ethyl alcohol diluted in autoclaved water) for two days. Ethanol concentration was then increased to 6% for two days, followed by an increase to 8% ethanol, the concentration presented for remainder of the experiment. Mice had continuous access to both water and ethanol channels throughout the experiment. Please refer to Figure 1 for a schematic of experimental timeline.

### Drug Administration

Following testing of baseline alcohol intake, we investigated the effects of a CRFR1 antagonist on alcohol consumption in the HM2 system. One day prior to drug administration, a habituation injection of vehicle (0.9% saline) was administered 30 min prior to the dark cycle. The following day, vehicle or 20 mg/kg CP-376,395 (diluted in 0.9% sterile saline, Batch 1B/226641, Tocris Bioscience, Minneapolis, MN USA) was administered 30 min prior to the dark cycle. All injections were administered at a volume of 10 ml/kg body weight. The dose of 20 mg/kg was selected based on previous work in our lab demonstrating its ability to decrease ethanol intake in singly-housed mice (Giardino and Ryabinin, 2013). In the socially-housed group, half of the animals (*n* = 2/cage) received drug while the other half (*n* = 2/cage) received vehicle, thus allowing administration in a “mixed” setting to more appropriately model treatment-assisted therapy for those undergoing AUD treatment. Intake, drinks, drink size, and channel entries for ethanol and water channels were monitored, where a drink represents an instance in which an animal entered the channel and consumed fluid, drink size represents the average volume (ml) consumed per drink, and a channel entry represents an instance in which an animal entered the channel, but did not consume fluid.

### Tissue Collection

Peak drinking and active time were noted 4 h into the dark cycle; therefore, animals in cohorts 2–5 were sacrificed 28 h after drug injection – 4 h into following day's dark cycle. Animals were sacrificed by CO_2_ asphyxiation and trunk blood was collected immediately and stored on ice for subsequent analysis. Brains were harvested prior to submerging in ice cold, 2% PFA. Following 24 h in 2% PFA, brains were transferred into 20% sucrose in PBS for 24 h, and then finally into 30% sucrose in PBS for at least 24 h prior to sectioning.

### Blood Ethanol Concentration (BEC)

Trunk blood was collected for the first cohort of animals 3 h into the dark cycle (27 h following drug injection) and BECs were analyzed using an Analox Analyzer (Analox Instruments, Luneburg, MO, USA) as previously described (Walcott and Ryabinin, 2020).

### Immunohistochemistry

Brains were sliced coronally at 30 μM using a Leica cryostat. Sections were then stained for FosB as previously described (Walcott and Ryabinin, 2020). Briefly, sections were incubated with primary antibody targeting FosB protein (rabbit anti-FosB, 1:27000, Lot GR214900-1, Abcam) followed by incubation in biotinylated goat anti-rabbit secondary (1:200, Vector Laboratories). Secondary antibody signal was amplified using a Vectastain ABC kit (Vector Laboratories) and visualized using a metal-enhanced 3-3'-diaminobenzidine (DAB) substrate kit (ThermoFisher Scientific). FosB immunoreactivity was analyzed in several known ethanol-sensitive brain regions (Vilpoux et al., 2009): the nucleus accumbens core and shell (NAcc, NAcs, AP + 1.4 to + 1.2), the central nucleus of the amygdala (CeA, AP −1.6 to −1.8), and the centrally-projecting Edinger-Westphal nucleus (EW, AP −3.6 to −3.8). Sections were imaged at 10x using a Leica DM40000 bright-field microscope and images analyzed using ImageJ's automated cell counting program. Approximate borders for each region were determined based on images provided in the Allen Mouse Brain Atlas (Allen Institute for Brain Science, 2004).

### Data Analysis

All data are presented as means ± standard error of the mean (SEM). All analyses were performed using SPSS software and graphs were constructed using GraphPad Prism software. Mixed-measure two-way ANOVAs were run to analyze the effect of social housing on baseline ethanol and water intake using the within-subjects factor of time and the between-subjects factor of housing condition. The dependent variable was ethanol or water intake in mls or g/kg, ethanol preference, number of ethanol or water drinks, drink size, and number of channel entries. Preference was calculated by dividing the amount of ethanol consumed by the total amount of fluid consumed over a 24 h period. Two-way ANOVAs were run to analyze the effect of CRFR1 antagonism on ethanol and water intake in individually- vs. socially-housed mice using the between-subject factors of drug treatment and housing condition for cumulative 3 h and 24 h data, whereas mixed-model three-way ANOVAs were used for hourly data analyses. The dependent variables were ethanol or water intake in g/kg, ethanol preference, number of ethanol or water drinks, drink size, and number of channel entries. BEC data were analyzed by student's *t*-test, comparing average BECs between individually- and socially-housed mice. FosB immunoreactivity was quantified by automated cell count in ImageJ, with positive cells identified by the detection of a significant increase in intensity above background, and FosB correlational data were analyzed using linear regression. Behavioral data from all animals were included in analyses (no outliers were excluded). A few data points for FosB analyses were excluded due to missing brain slices corresponding to the identical Bregma levels between animals. For complete, detailed statistics, please refer to the Supplementary Statistics Table.

## Results

### Cumulative 24-h Baseline Data

**Figure 2:**
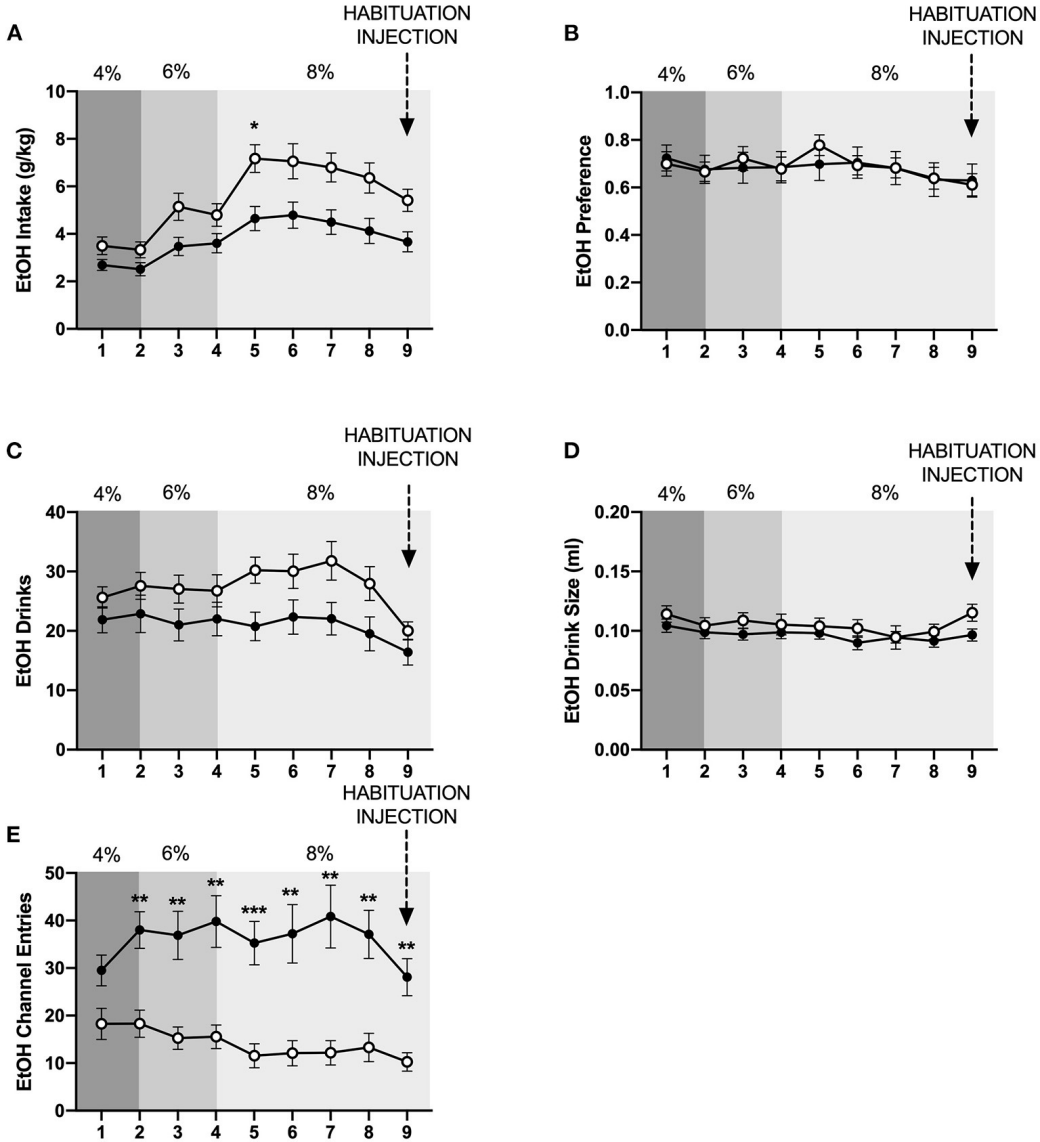
Cumulative 24 h baseline ethanol data. **(A)** Ethanol intake in g/kg. **(B)** EtOH preference. **(C)** Number of ethanol drinks. **(D)** Ethanol drink size in mls. **(E)** Ethanol channel entries. Overall, socially-housed mice consumed significantly more g/kg ethanol than individually-housed mice, and this higher level of consumption is due to a trend-level increase in number ethanol drinks and ethanol drink size in the socially-housed group. However, no differences in ethanol preference were observed. Additionally, individually-housed mice made significantly more non-consummatory ethanol channel entries than socially-housed mice. ^*^p <0.05, ^**^p <0.01, compared to opposite housing condition. Data represented as mean ± SEM. *n* = 24/group.

To characterize the effect of social housing on voluntary ethanol intake, ethanol intake in g/kg body weight, ethanol preference, number of ethanol drinks (instances in which an animal entered the channel and consumed ethanol), and ethanol drink size (volume consumed per drink) were compared between individually- and socially-housed subjects. Analysis of g/kg ethanol consumed revealed significant main effects of housing condition (*p* <0.01) and time (*p* <0.0001), as well as a significant interaction between these factors (*p* <0.01), with posthoc analysis showing that socially-housed mice consumed significantly more g/kg ethanol than individually-housed mice on day 5 (Figure 2A). This increased ethanol consumption in socially-housed mice is likely attributable to a combination of an increased number of ethanol drinks and increased drink size. Analysis of number of ethanol drinks revealed a significant main effect of time (*p* <0.0001) and a trend-level effect of housing condition (*p* = 0.05, Figure 2C), whereas analysis of ethanol drink size revealed a significant main effect of time (*p* <0.05) and a trend-level effect of housing condition (*p* = 0.07, Figure 2D). There were no interactions between housing and time for these two measures. These findings demonstrate that socially-housed mice consume significantly more ethanol than individually-housed mice, despite the fact that no difference in ethanol preference is observed between these groups (Figure 2B).

**Figure 3:**
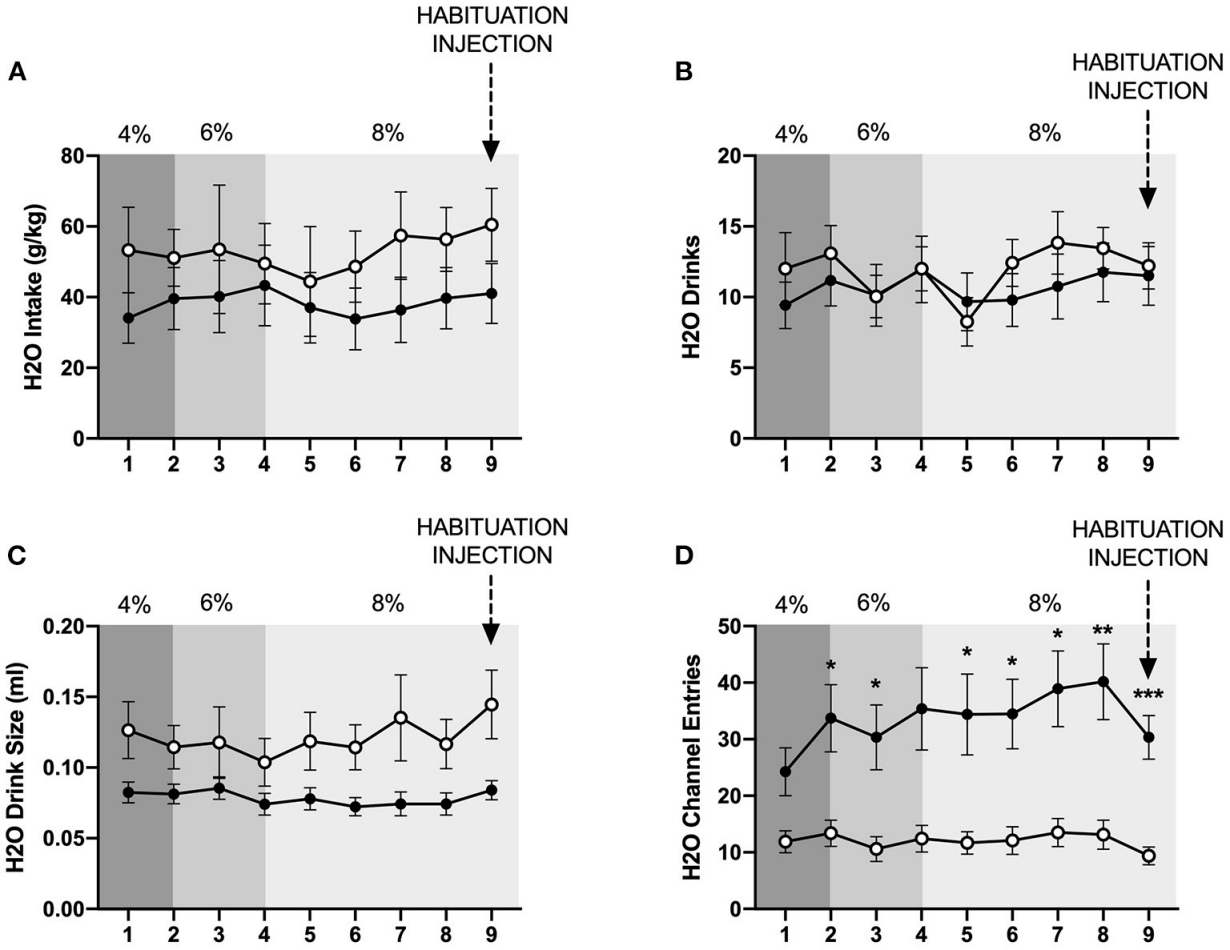
Cumulative 24 h baseline water data. **(A)** Water intake in g/kg. **(B)** Number of water drinks. **(C)** Water drink size. **(D)** Water channel entries. While no significant differences in g/kg water intake or number of water drinks were observed, socially-housed mice had significantly larger water drink size than individually-housed mice. As observed with the ethanol channel, individually-housed mice made significantly more non-consummatory water channel entries than socially-housed mice. ^*^p <0.05, ^**^p <0.01, ^***^p <0.001, compared to opposite housing condition. Data represented as mean ± SEM. *n* = 24/group.

In contrast to the findings for ethanol, mixed-measures two-way ANOVAs of g/kg water consumed and number of water drinks revealed no statistically significant differences between individually- and socially-housed subjects (Figures 3A,B). However, analysis of water drink size identified a significant main effect of housing condition (*p* <0.05), with socially-housed subjects consuming greater amounts of water per drink than individually-housed subjects (Figure 3C). This effect of social housing on drink size may explain why no significant difference in ethanol preference was detected between individually- and socially-housed mice, despite the fact that socially-housed mice consume more g/kg ethanol.

We also assessed the channel entries for ethanol and water, with channel entries representing instances in which a subject enters into a channel but does not consume any fluid. Two-way ANOVAs of both ethanol and water channel entries revealed significant main effects of housing condition (*p* <0.0001 and p <0.01), time (*p* <0.01 for both variables), and significant interactions between these factors (*p* <0.01, and *p* <0.05). Posthoc analysis of ethanol channel entries demonstrated that individually-housed mice made significantly more non-consummatory entries into the ethanol channel on days 2–9 (Figure 2E). Posthoc analysis of water channel entries demonstrated that individually-housed mice made significantly more non-consummatory entries into the water channel on days 2–3 and 5–9 (Figure 3D).

For complete statistics of 24 h cumulative data at baseline, please refer to Supplementary Statistics Table, and for ethanol and water intake results in mls, refer to Supplementary Figure 1.

### Hourly Baseline Data

#### EtOH Intake

**Figure 4:**
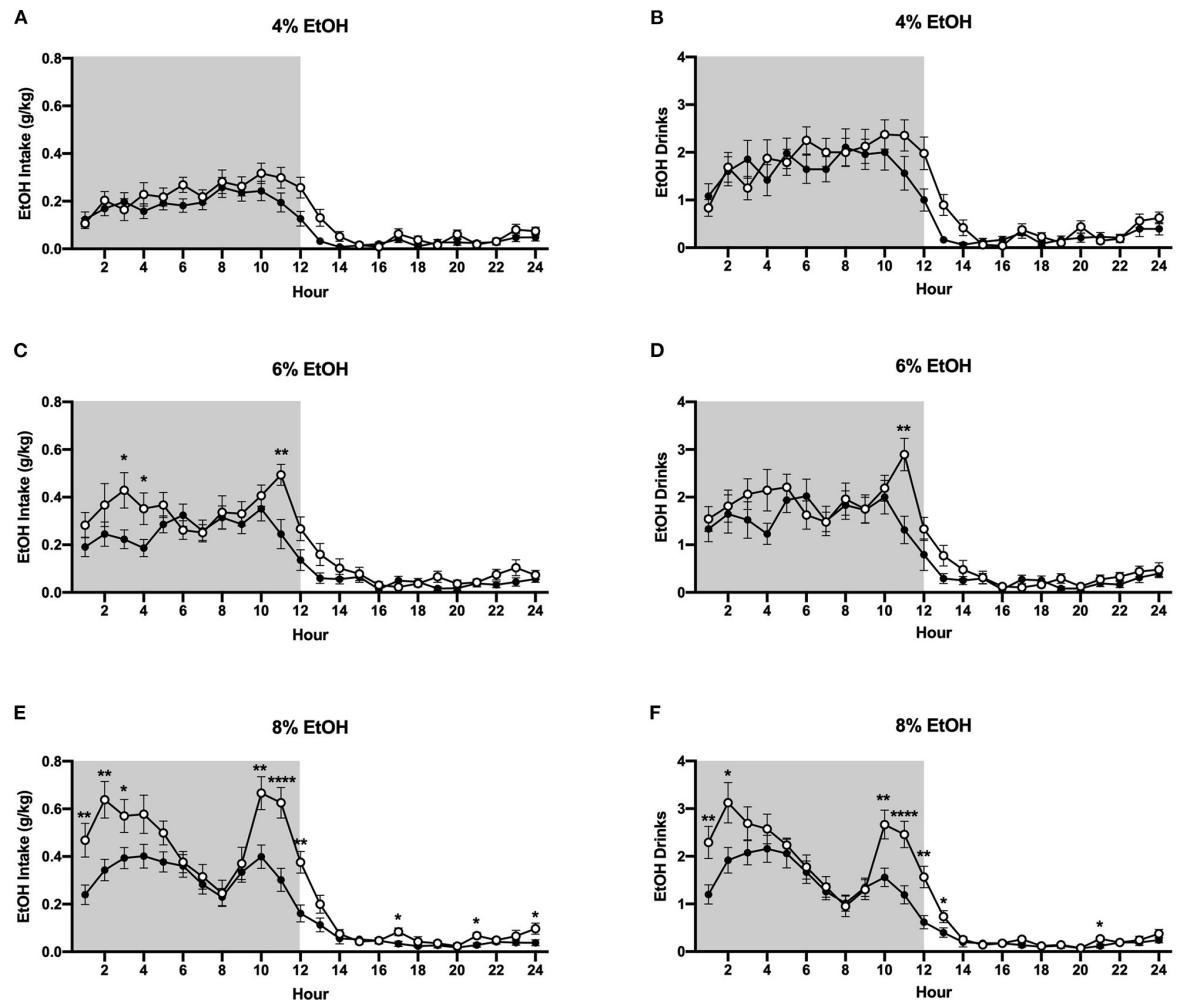
Hourly baseline ethanol data. **(A)** Ethanol intake in g/kg for 4% ethanol. **(B)** Number of ethanol drinks for 4% ethanol. **(C)** Ethanol intake in g/kg for 6% ethanol. **(D)** Number of ethanol drinks for 6% ethanol. **(E)** Ethanol intake in g/kg for 8% ethanol. **(F)** Number of ethanol drinks for 8% ethanol. ^*^p <0.05, ^**^p <0.01, ^****^p <0.0001, compared to opposite housing condition. Socially-housed mice consumed significantly more g/kg ethanol and had more ethanol drinks than individually-housed mice at the 6% and 8% ethanol concentrations. Data represented as mean ± SEM. *n* = 24/group. Shaded regions are representative of the dark cycle.

The HM2 system provides high temporal resolution of fluid consumption, allowing for assessment of ethanol and water intake at an hourly interval. Figure 4 data represent the mean values across each day that each ethanol concentration was available. At the 4% ethanol concentration, two-way ANOVA of g/kg ethanol intake revealed no effect of housing condition, nor an interaction between time and housing condition (Figure 4A), while at the 6% concentration, a significant interaction between time and housing condition (*p* <0.01) was observed (Figure 4C). Planned comparisons analysis demonstrated that socially-housed mice consumed significantly more g/kg ethanol than individually-housed mice at hours 3, 4, and 11 when 6% ethanol was available. At the 8% concentration, two-way ANOVA of g/kg ethanol intake identified significant main effects of time (*p* <0.0001) and housing condition (*p* <0.01), as well as a significant interaction between these factors (*p* <0.0001). Planned comparisons analyses revealed that socially-housed mice consumed significantly more g/kg ethanol than individually-housed mice at hours 1–3, 10–12, 17, 21, and 24 (Figure 4E). Overall, these data demonstrate that increased levels of g/kg ethanol intake in socially-housed mice are observed at the peak times of drinking and most strongly at the 8% ethanol concentration. For ethanol intake results in mls, refer to Supplementary Figure 2.

#### Ethanol Drinks

When the 4% ethanol concentration was available, two-way ANOVA of ethanol drinks revealed no effect of housing condition nor an interaction between time and housing condition (Figure 4B). At the 6% concentration, two-way ANOVA of ethanol channel entries revealed a main effect of time (*p* <0.0001) and a significant interaction between time and housing condition (*p* <0.05), with planned comparisons analysis revealing that individually-housed mice had significantly more ethanol drinks than socially-housed mice at hour 11 (Figure 4D). At the 8% ethanol concentration, two-way ANOVA of ethanol drinks revealed significant main effects of time (*p* <0.0001) and housing condition (*p* <0.05) and a significant interaction between these factors (*p* <0.0001). Planned comparisons analysis revealed that individually-housed animals had significantly more ethanol drinks than socially-housed animals at hours 1–2, 10–13, and 21 (Figure 4F). These findings show that the increased ethanol intake observed in socially-housed mice is driven, at least in part, by an increased number of ethanol drinks.

#### Water Intake

**Figure 5:**
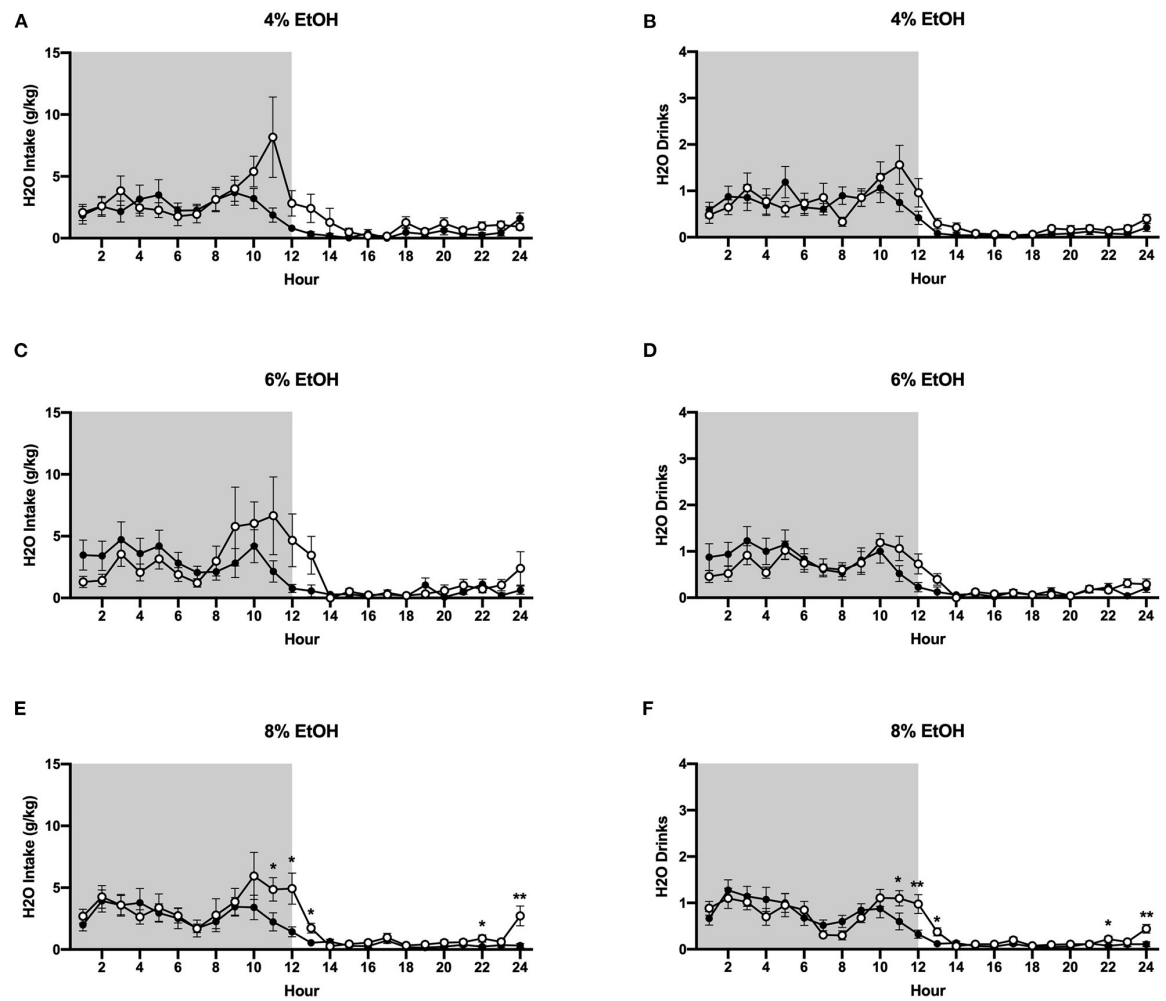
Hourly baseline water data. **(A)** Water intake in g/kg when 4% ethanol was available. **(B)** Number of water drinks when 4% ethanol was available. **(C)** Water intake in g/kg when 6% ethanol was available. **(D)** Number of water drinks when 6% ethanol was available. **(E)** Water intake in g/kg when 8% ethanol was available. **(F)** Number of water drinks when 8% ethanol was available. Socially-housed mice had significantly higher water intake and number of water drinks than individually-housed mice, but only at the 8% ethanol concentration. ^*^p <0.05, ^**^p <0.01, compared to opposite housing condition. Data represented as mean ± SEM. *n* = 24/group. Shaded regions are representative of the dark cycle.

In contrast to ethanol consumption results, analysis of g/kg water intake when 4% ethanol was available revealed a significant interaction between time and housing condition (*p* <0.01), although planned comparisons analysis revealed no differences in g/kg water intake between groups at specific timepoints (Figure 5A). Similarly, when the ethanol concentration was increased to 6%, two-way ANOVA of g/kg water intake a significant interactions between time and housing condition (*p* <0.05), although planned comparisons analysis revealed no differences between socially- and individually-housed mice at any timepoint (Figure 5C). However, when 8% ethanol was available, analysis of g/kg water intake identified a significant interaction between time and housing condition (*p* <0.01), with planned comparisons analysis demonstrating that socially-housed mice consumed significantly more g/kg water than individually-housed mice at hours 11–13, 22, and 24 (Figure 5E). These data suggest that the increased fluid intake in socially-housed mice is not specific to ethanol, in that socially-housed subjects also consumed more water than individually-housed mice, an effect that reached posthoc statistical significance at the 8% concentration. For water intake results in mls, refer to Supplementary Figure 2.

#### Water Drinks

Two-way ANOVA of water drinks at the 4 and 6% ethanol concentrations revealed significant main effects of time (*p* <0.0001 for both measures) and trend-level interactions between time and housing condition (*p* = 0.05, *p* = 0.06, Figures 5B,D). However, when the ethanol concentration was increased to 8%, two-way ANOVA of water drinks revealed a significant interaction between time and housing condition (*p* <0.0001), with planned comparisons analysis revealing that socially-housed mice had significantly more water drinks than individually-housed mice at hours 11–13, 22, and 24 (Figure 5F). Taken together, these data suggest that the observed increased g/kg water intake in socially-housed mice is, at least in part, attributable to an increased number of water drinks.

#### Ethanol and Water Channel Entries

**Figure 6:**
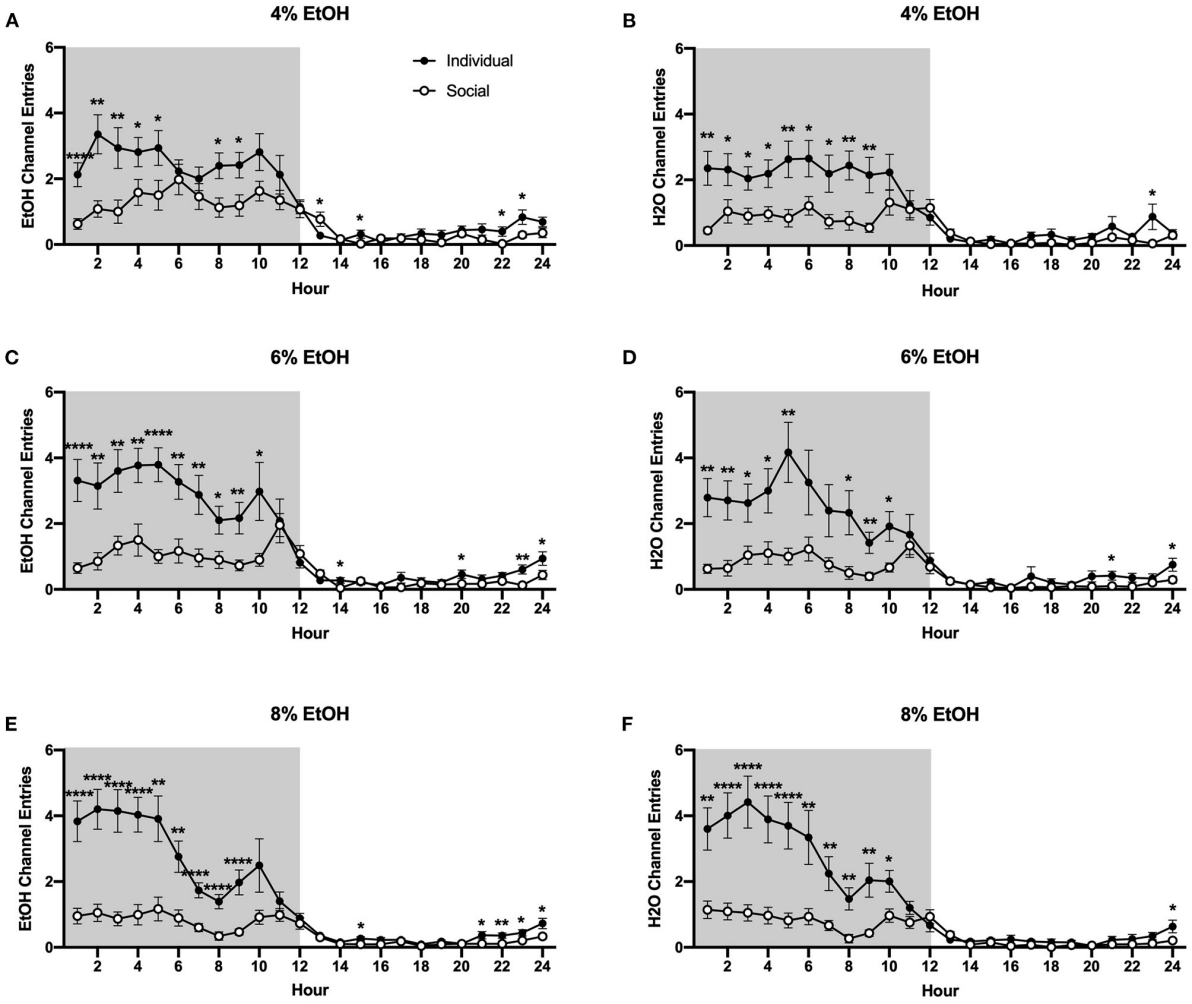
Hourly baseline channel entries. **(A)** Ethanol and **(B)** water channel entries when 4% ethanol was available. **(C)** Ethanol and **(D)** water channel entries when 6% ethanol was available. **(E)** Ethanol and **(F)** water channel entries when 8% ethanol was available. For both ethanol and water channels, and across all ethanol concentrations presented, individually-housed mice made significantly more non-consummatory channel entries than socially-housed mice. ^*^p <0.05, ^**^p <0.01, ^****^p <0.0001, compared to opposite housing condition. Data represented as mean ± SEM. *n* = 24/group. Shaded regions are representative of the dark cycle.

Interestingly, as for the 24 h data, we found that individually-housed mice made significantly more non-consummatory ethanol and water channel entries, regardless of the ethanol concentration that was available. Two-way ANOVA of ethanol channel entries at the 4% concentration revealed a significant main effect of housing condition (*p* <0.01) and a significant interaction between housing condition and time (*p* <0.0001), with planned comparisons analysis demonstrating that individually-housed mice made significantly more ethanol channel entries than socially-housed mice at hours 1–5, 8–9, 15, and 22–23, but significantly less entries at hour 13 (Figure 6A). Similarly, analysis of water channel entries at this ethanol concentration revealed a significant main effect of housing condition (*p* <0.01), as well as a significant interaction between housing condition and time (*p* <0.0001). Planned comparisons demonstrated that individually-housed mice made significantly more non-consummatory water channel entries than socially-housed mice at hours 1–9 and 23 (Figure 6B). When the ethanol concentration was increased to 6%, analysis of ethanol channel entries also revealed a significant main effect of housing condition (*p* <0.0001) and a significant interaction between housing condition and time (*p* <0.0001), with planned comparisons analysis demonstrating that individually-housed mice made significantly more ethanol channel entries than socially-housed males at hours 1–10, 14, 20, and 23–24 (Figure 6C). Water channel entries at this concentration also showed a significant main effect of housing condition (*p* <0.01) and a significant interaction between housing condition and time (*p* <0.0001), with planned comparisons analysis demonstrating that individually-housed mice completed more water channel entries than socially-housed mice at hours 1–5, 8–10, 19, 21, and 24 (Figure 6D). As observed in the previous two concentrations, when 8% ethanol was available, analysis of ethanol channel entries revealed a significant main effect of housing condition (*p* <0.0001) and a significant interaction between housing condition and time (*p* <0.0001), with planned comparisons analysis demonstrating that individually-housed mice made significantly more ethanol channel entries than socially-housed mice at hours 1–9, 15, and 21–24 (Figure 6E). Similarly, analysis of water channel entries at this concentration also revealed a significant main effect of housing condition (*p* <0.0001) and a significant interaction between housing condition and time (*p* <0.0001), with planned comparisons analysis demonstrating that individually-housed mice made significantly more water channel entries than socially-housed mice at hours 1–10 and 24 (Figure 6F).

#### Ethanol Preference, Ethanol and Water Drink Size

Unfortunately, we were unable to conduct analyses for ethanol preference for hourly data due to the high number of missing data points on each day of drinking (the result of animals not consuming fluid every hour). Similarly, we were unable to conduct hourly analyses for ethanol or water drink size to the high number of missing data points on each day of drinking (also the result of animals not consuming fluid every hour). For graphs of these data, please refer to Supplementary Figure 3.

### Effect of CRFR1 Antagonism

#### Cumulative 24-h Data

**Figure 7:**
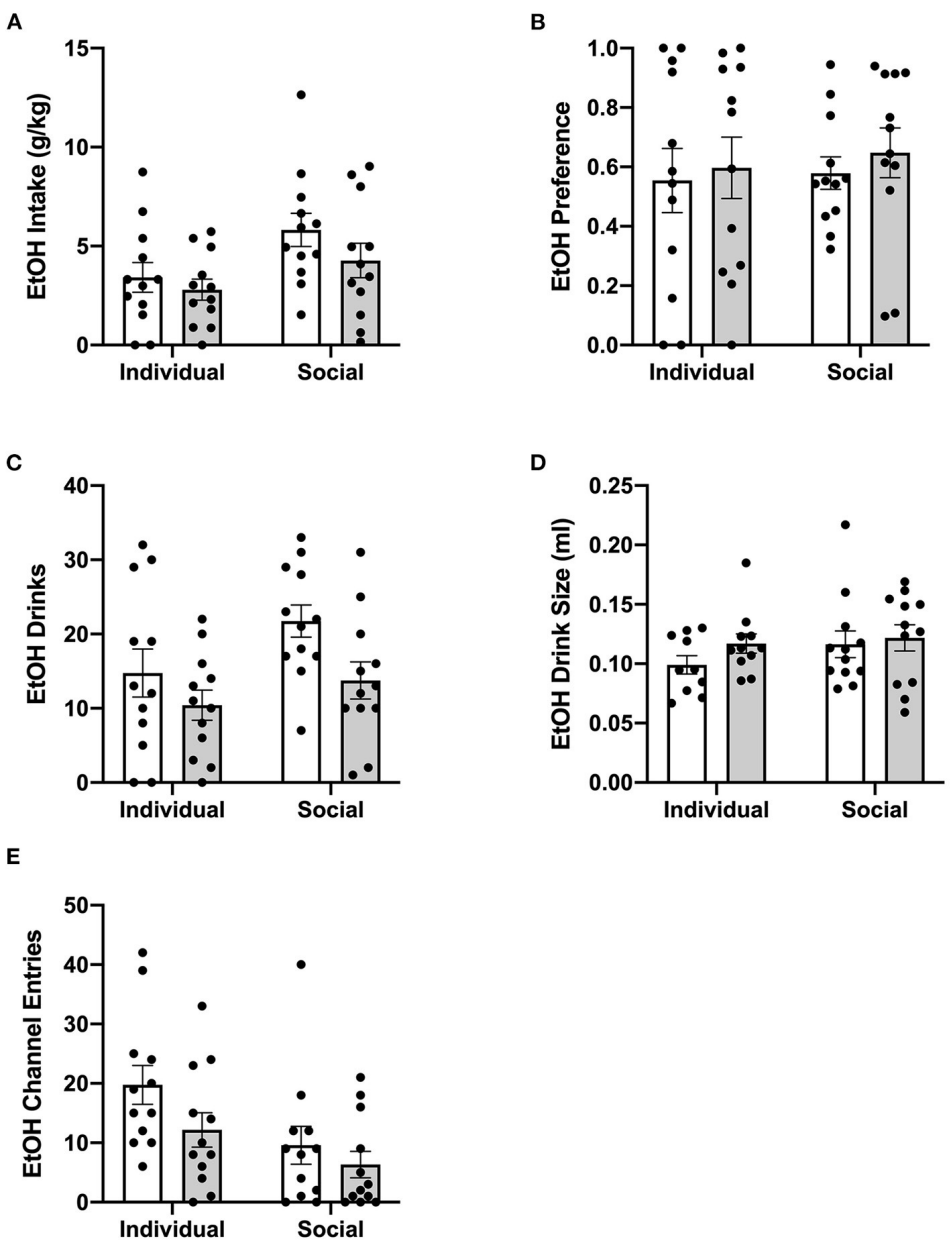
Twenty four hour cumulative ethanol data post-CRFR1 antagonism. **(A)** Ethanol intake in g/kg. **(B)** Ethanol preference. **(C)** Number of ethanol drinks. **(D)** Ethanol drink size in mls. **(E)** Ethanol channel entries. While no effects of CRFR1 antagonism on g/kg ethanol intake, ethanol preference, or ethanol drink size were observed, this treatment did cause a significant decrease in number of ethanol drinks across both housing conditions. This treatment also selectively decreased non-consummatory channel entries in individually-housed mice. Data represented as mean ± SEM. *n* = 12/group.

Previously, suppressive effects of CP-376,395 administration on water and ethanol intake were observed 3 h, but not 24 h, post-treatment in mice housed individually in standard, shoebox cages (Potretzke et al., 2020). To identify whether CRF1R antagonism would affect ethanol intake in animals housed in the HM2 system, ethanol intake, ethanol preference, number of ethanol drinks (instances in which an animal entered the channel and consumed ethanol), and ethanol drink size (volume consumed per drink) were compared between individually- and socially-housed subjects treated with vehicle or CP-376,395 at 24 hours post-treatment (for mls intake data, refer to Supplementary Figure 4). Two-way ANOVA of g/kg ethanol intake revealed no effect of CRFR1 antagonism (Figure 7A). However, two-way ANOVA of ethanol drinks revealed significant main effects of housing condition (*p* <0.05) and of CRFR1 antagonism (*p* <0.01, Figure 7C), though ethanol drink size was unaffected (Figure 7D). Similarly, no effect of CRFR1 antagonism on ethanol preference was observed (Figure 7B).

**Figure 8:**
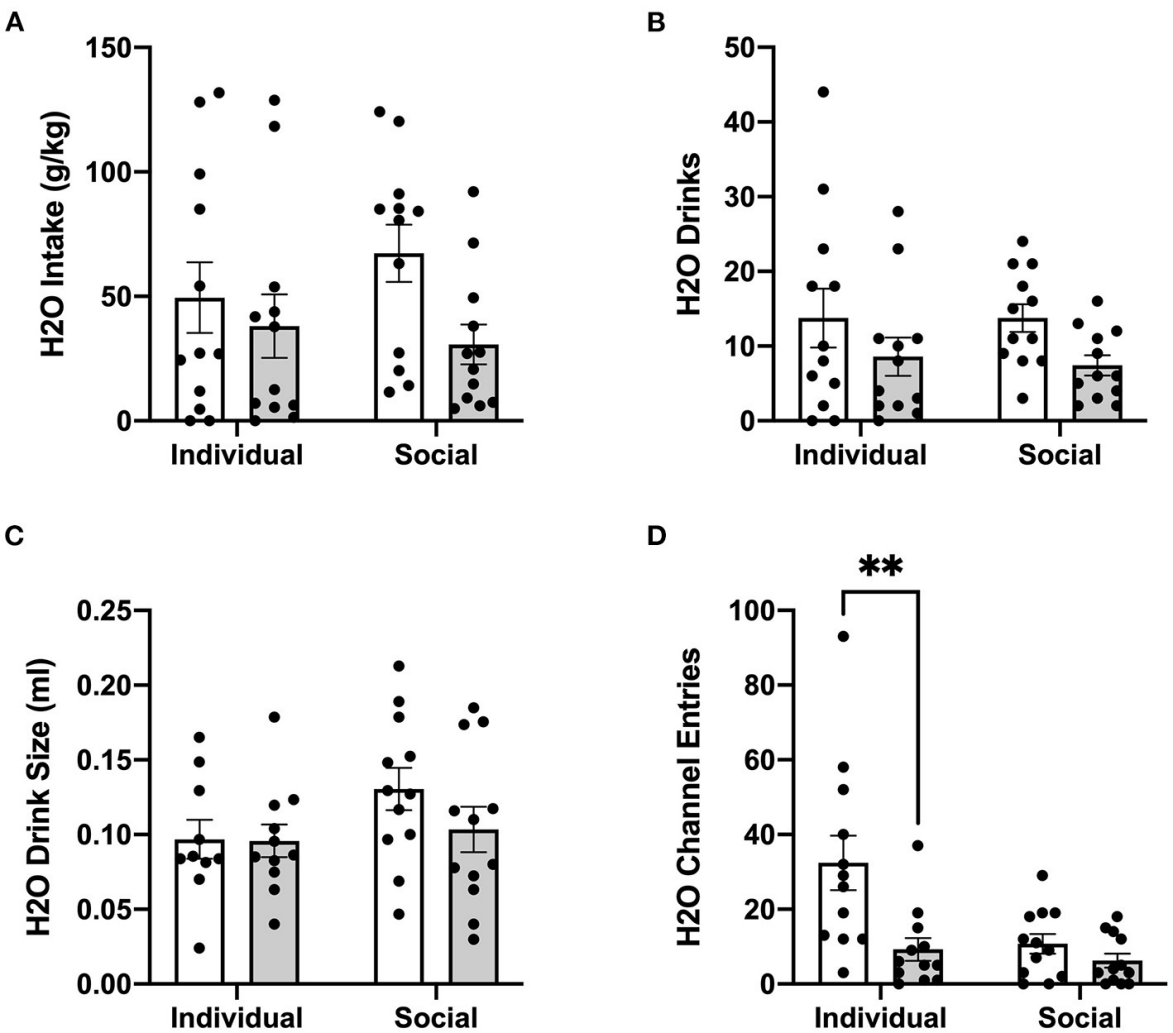
24 h cumulative water data post-CRFR1 antagonism. **(A)** Water intake in g/kg. **(B)** Number of water drinks. **(C)** Water drink size in mls. **(D)** Water channel entries. ^**^p <0.01, compared to CP-376,39- treated individually-housed mice. While water drink size was unaffected by CRFR1 antagonism, this treatment caused significant decreases in g/kg water intake and number of water drinks across both housing conditions. As observed with the ethanol channel, this treatment also caused a selective decrease in non-consummatory channel entries in individually-housed mice. Data represented as mean ± SEM. *n* = 12/group.

In contrast to ethanol, analysis of g/kg water intake at 24 h post-treatment identified significant main effect of CRFR1 antagonism (*p* <0.05), but no differences between individually- and socially-housed mice (Figure 8A). This finding also extended to water drinks, with a significantly lower number of drinks following the CRFR1 antagonist (*p* <0.05) across both housing conditions (Figure 8B). Water drink size was unaffected by drug treatment or housing condition (Figure 8C).

Lastly, we assessed the effect of CRFR1 antagonism on number of (non-consummatory) channel entries for individually- and socially-housed mice. Consistently with what was observed at baseline, two-way ANOVA of ethanol channel entries revealed a significant main effect of housing condition (*p* <0.01, Figure 7E), with individually-housed mice entering the channel frequently more than socially-housed mice. Notably, though, a trend-level effect of CRFR1 antagonism on ethanol channel entries was also observed (*p* = 0.07). Results from two-way ANOVA of water channel entries also revealed significant main effects of housing (*p* <0.01), treatment (*p* <0.01), and a significant interaction between these factors (*p* <0.05), with posthoc analysis demonstrating that the CRFR1 antagonism-induced decrease in water channel entries only reached significance for individually-housed mice (*p* <0.01, Figure 8D).

#### Hourly Data

**Figure 9:**
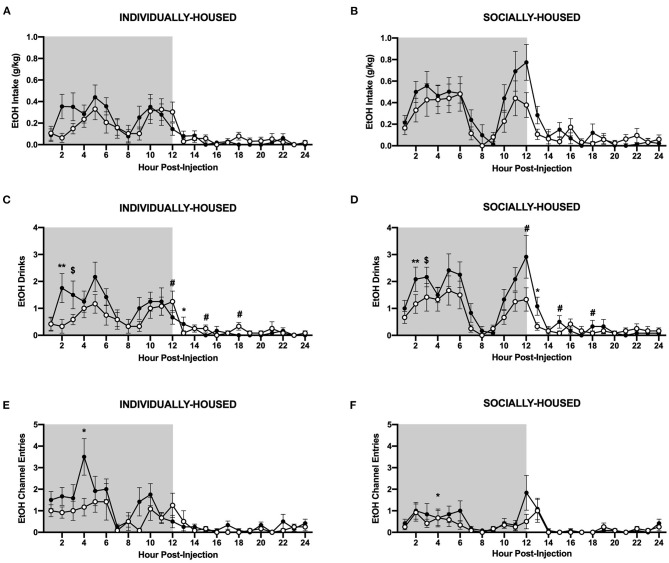
Hourly ethanol data post-CRFR1 antagonism. **(A)** Ethanol intake in g/kg for individually-housed mice. **(B)** Ethanol intake in g/kg for socially-housed mice. **(C)** Number of ethanol drinks for individually-housed mice. **(D)** Number of ethanol drinks for socially-housed mice. **(F)** Ethanol channel entries for individually-housed mice. **(E)** Ethanol channel entries for socially-housed mice. As observed in the cumulative 24 h data, no effect of CRFR1 antagonism on g/kg ethanol intake was observed, but this treatment did cause a time-dependent decrease in the number of ethanol drinks in both housing conditions. Additionally, CRFR1 antagonism caused a trend-level decrease in non-consummatory channel entries, and this decrease is likely driven by the individually-housed group. $*p* = 0.05, ^*^p <0.05, ^**^p <0.01, compared to vehicle-treated mice across housing condition. #*p* <0.05 represents interaction between housing condition and CRFR1 antagonism at specific timepoint. Data represented as mean ± SEM. *n* = 12/group. Shaded regions are representative of the dark cycle.

As with baseline data, we also analyzed the effects of CRFR1 antagonism on water and ethanol measures at an hourly interval. Data were plotted separately for visualization purposes only. Three-way ANOVA of g/kg ethanol intake revealed a significant main effect of housing condition (*p* <0.05), but no effect of CRFR1 antagonism, nor any significant interactions involving this treatment (Figures 9A,B). However, analysis of ethanol drinks revealed significant main effects of housing condition (*p* <0.05) and CRFR1 antagonism (p <0.05), and a significant interaction between time and CRFR1 antagonism (*p* <0.05, Figures 9C,D). Planned comparisons analysis revealed a significant effect of CRFR1 antagonism in individually- and socially-housed mice 2 h post-treatment (*p* <0.01), a trend-level effect of CRFR1 antagonism in both groups 3 h post-treatment (*p* = 0.05), and a significant effect of housing condition, regardless of treatment, at 9 h post-treatment (*p* <0.05). This analysis also revealed a significant effect of housing condition (*p* <0.05) and a significant interaction between housing condition and CRFR1 antagonism (*p* <0.05) at 12 h post-treatment, with antagonist-treated subjects having more ethanol drinks relative to controls in the individually-housed group and less ethanol drinks relative to controls in the socially-housed group. At 13 h post-treatment, a trend-level effect of housing condition (*p* = 0.05) and a significant effect of CRFR1 antagonism were observed (*p* <0.05), with socially-housed mice having more ethanol drinks than individually-housed subjects, and CRFR1 antagonist-treated mice having less drinks than vehicle-treated subjects. At 15 h and 18 h post-treatment, a significant interaction between housing condition and CRFR1 antagonism was observed (*p* <0.05 for both timepoints), with antagonist-treated subjects having more ethanol drinks relative to controls in the individually-housed group and less ethanol drinks relative to controls in the socially-housed group.

**Figure 10:**
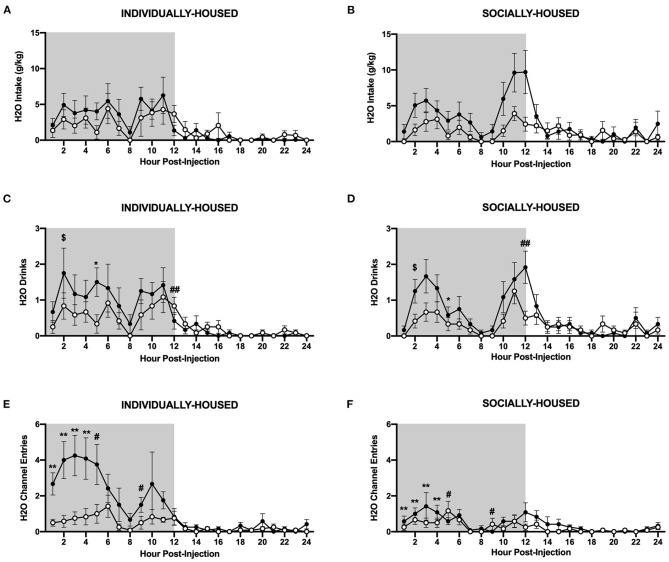
Hourly water data post-CRFR1 antagonism. **(A)** Water intake in g/kg for individually-housed mice. **(B)** Water intake in g/kg for socially-housed mice. **(C)** Number of water drinks for individually-housed mice. **(D)** Number of water drinks for socially-housed mice. **(E)** Water channel entries for individually-housed mice. **(F)** Water channel entries for socially-housed mice. CRFR1 antagonism caused decreases in g/kg water intake and number of water drinks for both socially- and individually-housed mice. This treatment also suppressed non-consummatory channel entries in both groups. $*p* = 0.05, ^*^p <0.05, ^**^p <0.01, compared to vehicle-treated mice across housing condition. #*p* <0.05 and ##*p* <0.01 represent interaction between housing condition and CRFR1 antagonism at specific timepoint. Data represented as mean ± SEM. *n*=12/group. Shaded regions are representative of the dark cycle.

In contrast to ethanol, three-way ANOVA of g/kg water intake revealed a significant main effect of CRFR1 antagonism (*p* <0.05, Figures 10A,B). Three-way ANOVA of water drinks showed a significant main effect of CRFR1 antagonism (p <0.05) and a significant interaction between time and CRFR1 antagonism (*p* <0.05, Figures 10C,D). Planned comparisons analysis of water drinks revealed a significant effect of housing condition at 1 h post-CRFR1 antagonism. A trend-level effect of CRFR1 antagonism was then observed across both housing conditions 2 h post-treatment (*p* = 0.05), with a significant effect observed 5 h post-treatment (*p* <0.05). A significant effect of housing was again seen at 9 h post-treatment (*p* <0.01). Lastly, at 12 h post-treatment, a significant interaction between CRFR1 antagonism and housing condition was observed, in that antagonist-treated subjects had more water drinks than controls in the individually-housed group, but less water drinks than controls in the socially-housed group (*p* <0.01). For hourly mls intake data, refer to Supplementary Figure 4.

Interestingly, there were also effects of CRFR1 antagonism on non-consummatory channel entries for individually-housed mice. Specifically, three-way ANOVA of ethanol channel entries revealed a significant main effect of housing condition (*p* <0.01), a trend-level effect of CRFR1 antagonism (*p* = 0.07), and a significant interaction between time, housing condition, and CRFR1 antagonism (*p* <0.05, Figures 9E,F). Planned comparisons analysis revealed a significant main effect of housing condition (*p* <0.01) at 4 h post-treatment, with individually-housed mice completing more ethanol channel entries than socially-housed mice. A significant main effect of CRFR1 antagonism (*p* <0.05) and a significant interaction between CRFR1 antagonism and housing condition (*p* <0.05) were also observed at this timepoint, in that CRFR1 antagonism only caused a decrease in ethanol channel entries in individually-housed mice receiving this treatment. At 9 h post-treatment, a significant interaction between housing condition and CRFR1 antagonism was observed (*p* <0.05), with CRFR1 antagonism causing a decrease in ethanol channel entries in individually-housed mice only. At 10 h post-treatment, however, only a main effect of housing condition was observed (*p* <0.05), with individually-housed mice making more ethanol channel entries than socially-housed subjects.

Similarly, three-way ANOVA of water channel entries revealed significant main effects of housing condition (*p* <0.01), CRFR1 antagonism (*p* <0.01), and a significant interaction between time, housing condition, and CRFR1 antagonism (*p* <0.0001, Figures 10E,F). Planned comparisons analysis revealed significant main effects of housing condition (*p* <0.01) and CRFR1 antagonism (*p* <0.01) and a significant interaction between these factors (*p* <0.05) at 1 h post-treatment. Similar findings were observed at the 2 h timepoint as well, with planned comparisons analysis revealing main effects of housing condition (*p* <0.05) and CRFR1 antagonism (p <0.01), and a significant interaction between these factors (p <0.05). Again, at 3 and 4 h post-treatment, main effects of housing condition (*p* <0.05 for both timepoints) and CRFR1 antagonism (*p* <0.01, for both timepoints) were observed, with the addition of a trend-level interaction between housing condition and CRFR1 antagonism (*p* = 0.05) for the 4 h timepoint. At 5 h post-treatment, a main effect of housing condition (*p* <0.05) and a significant interaction between housing condition and CRFR1 antagonism (*p* <0.05) were observed, in that antagonist-treated subjects exhibited decreased water channel entries compared to controls in the individually-housed group and increased entries compared to controls in the socially-housed group. At the 6 h timepoint, analysis showed that individually-housed mice made significantly more water channel entries than socially-housed subjects. At 9 h post-treatment, a main effect of housing condition (*p* <0.05) and an interaction between housing condition and CRFR1 antagonism (*p* <0.05), with antagonist-treated subjects exhibiting decreased water channel entries compared to controls in the individually-housed group and increased entries compared to controls in the socially-housed group. At 18 h post-treatment, analysis demonstrated that individually-housed mice, again, made significantly more water channel entries than socially-housed mice (*p* <0.05).

Overall, these data demonstrate that when analyzed at the hourly interval, CRFR antagonism did not significantly affect ethanol intake, even though this treatment caused a transient decrease in number of ethanol drinks. In contrast, CRFR1 antagonism decreased water intake and drinks. This treatment also caused a decrease in non-consummatory ethanol and water channel entries, but this decrease was specific to individually-housed mice.

#### Cumulative 3 h Data

**Figure 11:**
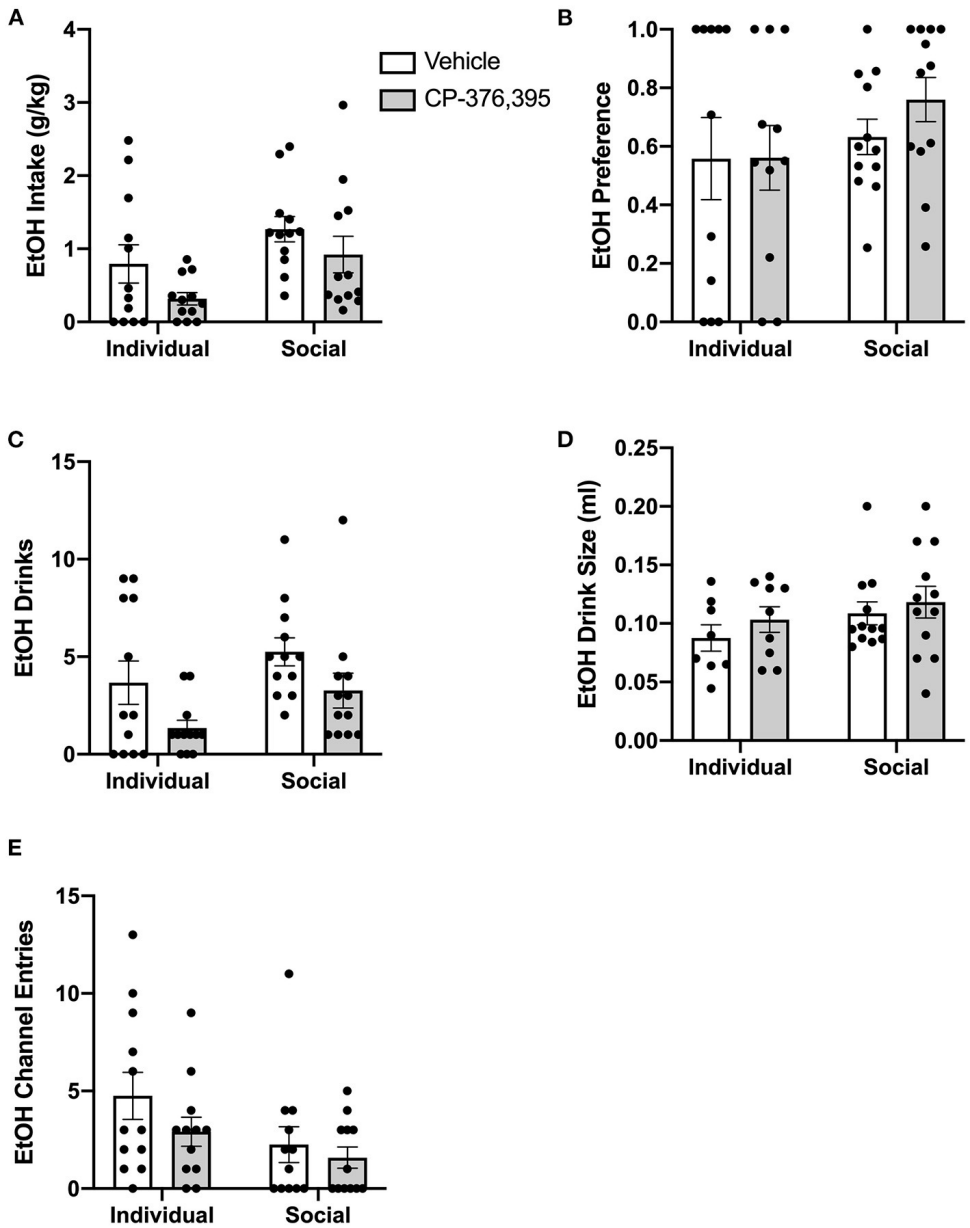
Three hour cumulative ethanol data post-CRFR1 antagonism. **(A)** Ethanol intake in g/kg. **(B)** Ethanol preference. **(C)** Number of ethanol drinks. **(D)** Ethanol drink size in mls. **(E)** Ethanol channel entries. During the first 3 h post-treatment, CRFR1 antagonism caused decreases in g/kg ethanol intake and the number of ethanol drinks across both housing conditions, with ethanol preference, drink size, and channel entries remaining unaffected. Data represented as mean ± SEM. *n* = 12/group.

Due to the complex patterns of intake, drinks, and entries observed in the hourly data analyses outlined above, and to make our studies comparable to previous studies devoid of capabilities to precisely measure intake at short time intervals, we then conducted analyses on cumulative data from the first 3 h post-treatment. Two-way ANOVA of g/kg ethanol intake revealed a main effect of housing condition for g/kg ethanol intake (*p* <0.05), as well as a trend-level effect of CRFR1 antagonism (*p* = 0.05, Figure 11A). This CRFR1 antagonism-induced decrease in ethanol intake is attributable to a decreased number of ethanol drinks, with two-way ANOVA of this measure revealing significant effects of housing condition (*p* <0.05) and CRFR1 antagonism (*p* <0.01, Figure 11C), with ethanol drink size unaffected by this treatment or by housing condition (Figure 11D). Ethanol preference did not differ as a product of housing condition or CRFR1 antagonism (Figure 11B).

**Figure 12:**
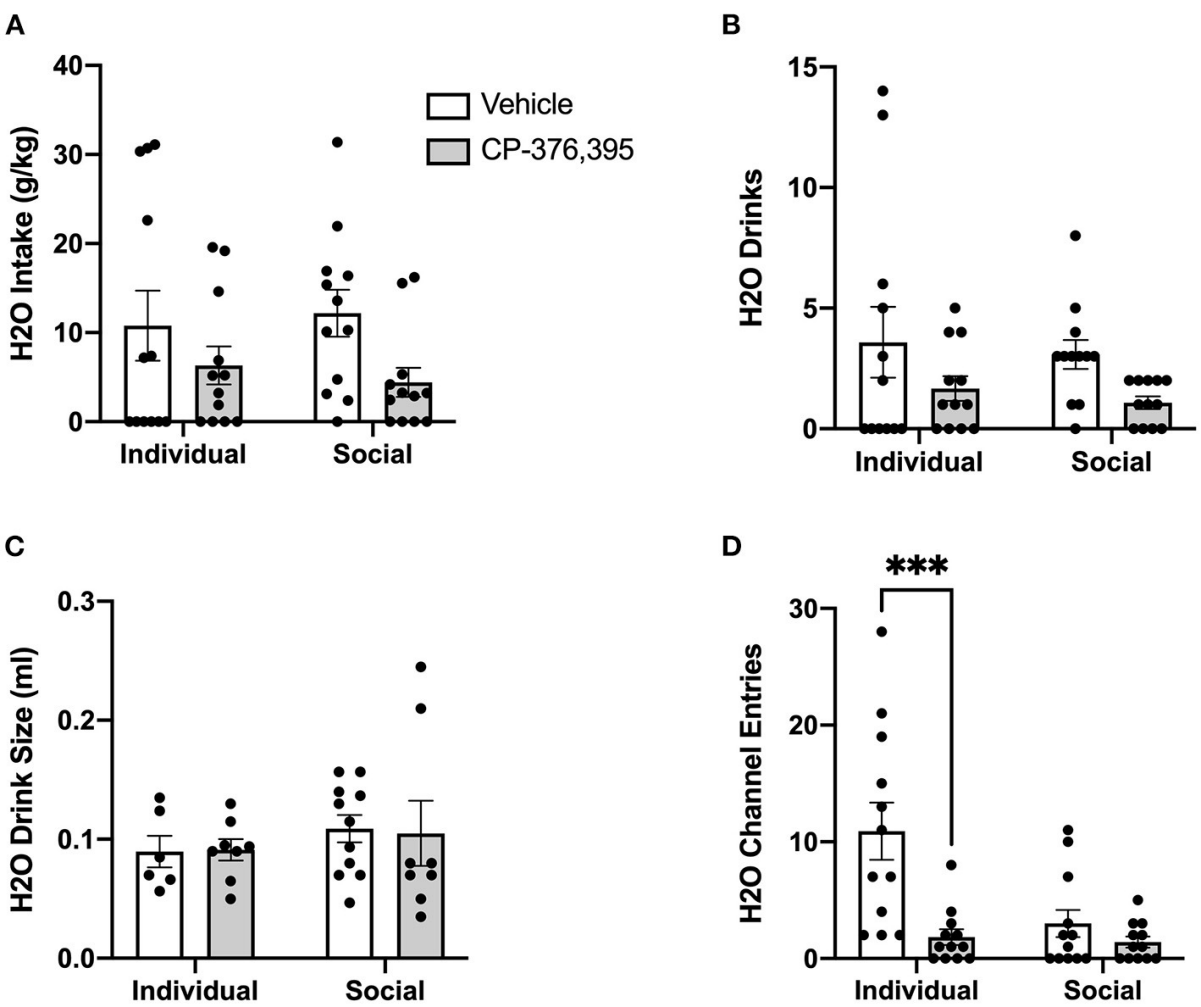
Three hour cumulative water data post-CRFR1 antagonism. **(A)** Water intake in mls. **(B)** Water intake in g/kg. **(C)** Number of water drinks. **(D)** Water drink size in mls. **(E)** Water channel entries. During the first 3 h post-treatment, CRFR1 antagonism caused decreases in g/kg water intake and the number of water drinks across both housing conditions, with water drink size remaining unaffected. This treatment also caused a selective decrease in non-consummatory channel entries for the individually-housed group. Data represented as mean ± SEM. *n* = 12/group.

Water intake in g/kg was also significantly decreased following CRFR1 antagonism, though intake levels did not differ between individually- and socially-housed mice (Figure 12A). As with ethanol intake, this treatment-induced decrease in water intake is due to a decrease in water drinks, with two-way ANOVA revealing a main effect of CRFR1 antagonism (p <0.05), despite no differences across housing conditions (Figure 12B). In contrast, water drink size was not affected by CRFR1 antagonism or housing condition (Figure 12C). For ethanol and water intake data in mls, refer to Supplementary Figure 4.

As we observed with the cumulative 24 h and hourly data, analysis of ethanol channel entries revealed a main effect of housing condition (*p* <0.05) but no effect of CRFR1 antagonism (Figure 11E). In contrast, analysis of water channel entries revealed main effects of housing condition (*p* <0.01) and CRFR1 antagonism (*p* <0.01) and a significant interaction between these factors, with posthoc analysis showing that CRFR1 antagonism selectively decreased water channel entries for individually-housed mice (*p* <0.001, Figure 12D).

Thus, analysis of cumulative 3 h data revealed suppressive effects of CRFR1 antagonism on measures of ethanol intake irrespective of housing and selective effects of this treatment on non-consummatory water channel entries in individually-housed mice.

### Relationships Between Ethanol Intake, BECs, and FosB Immunoreactivity

#### Ethanol Intake and Blood Ethanol Concentrations (BECs)

Blood ethanol concentrations (BECs) were analyzed for the first cohort of animals (*n* = 8 socially-housed and *n* = 4 individually-housed mice). These analyses revealed a range in BEC levels from 5.97 to 77.58 mg/dL, though no difference was observed between individually- and socially-housed mice (Supplementary Figure 7). However, this is not surprising given the lack of observed difference in g/kg ethanol intake as well (Supplementary Figure 7A). While the higher values confirmed that alcohol consumption results in physiologically relevant BECs in certain animals at some point of the drinking experiment, we needed another measure of physiological effects resulting from alcohol consumption. Therefore, we analyzed FosB immunoreactivity in the subsequent four cohorts as a marker for prolonged changes in neural activity across four brain regions known to be activated following ethanol exposure.

#### Ethanol Intake and FosB Immunoreactivity

**Figure 13:**
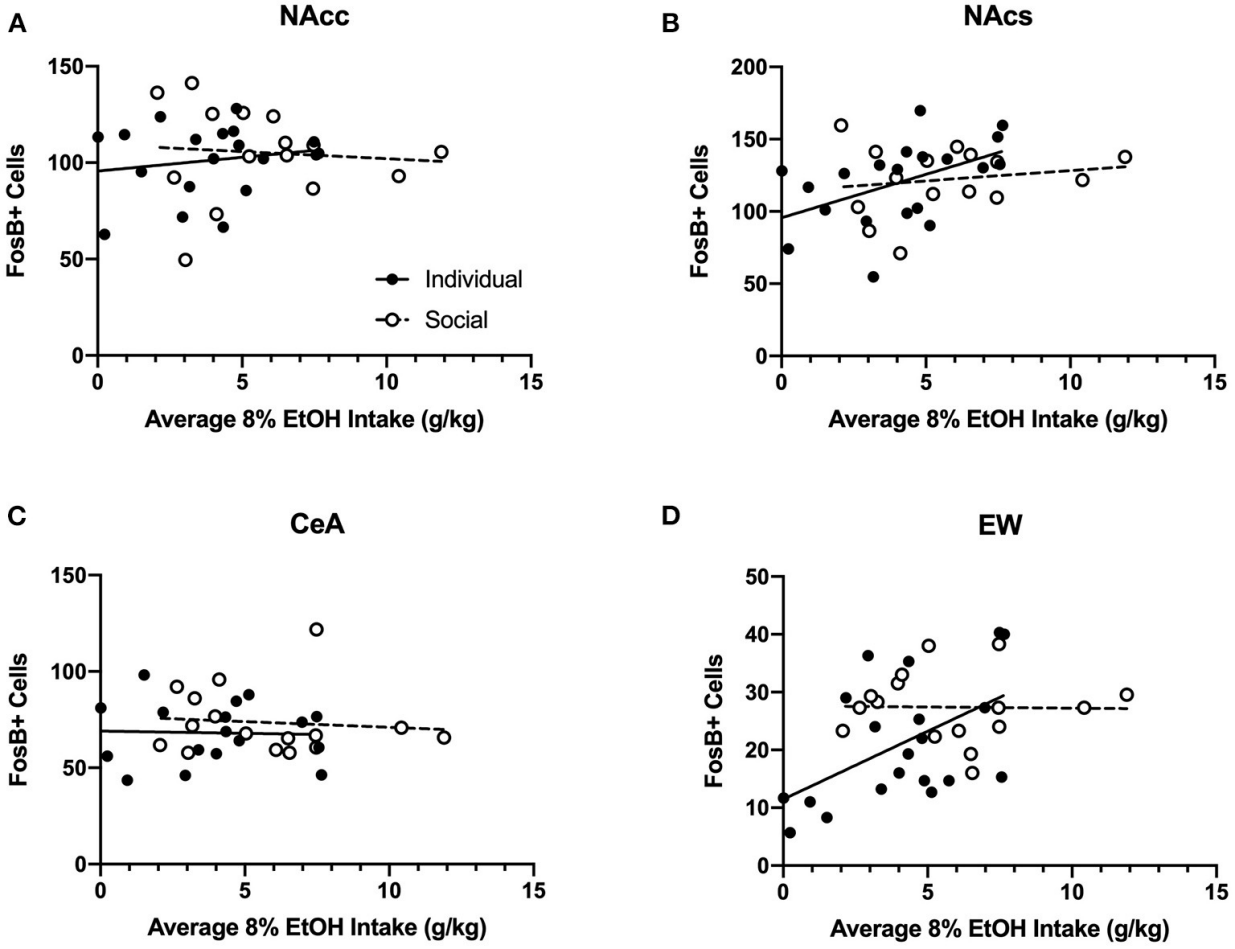
Correlations between average baseline g/kg ethanol intake and FosB-positive cells in various ethanol-responsive brain regions. Ethanol data in g/kg represent average intake across all days of baseline 8% ethanol availability. **(A)** Intake correlation with FosB-positive cells in the nucleus accumbens core (NAcc). For individually-housed mice, *r*^2^ = 0.03, *p* = 0.47, *n* = 20. For socially-housed mice, *r*^2^ = 0.007, *p* = 0.76, *n* = 15. **(B)** Intake correlation with FosB-positive cells in the nucleus accumbens shell (NAcs). For individually-housed mice, *r*^2^ = 0.24, *p* <0.05, *n* = 19. For socially-housed mice, *r*^2^ = 0.03, *p* = 0.54, *n* = 15. **(C)** Intake correlation with FosB- positive cells in the central amygdala (CeA). For individually-housed mice, *r*^2^ = 0.001, *p* = 0.89, *n* = 18. For socially-housed mice, *r*^2^ = 0.009, *p* = 0.72, *n* = 16. **(D)**. Intake correlation with FosB- positive cells in the centrally-projecting Edinger-Westphal nucleus (EW). For individually-housed mice, *r*^2^ = 0.27, *p* <0.05, *n* = 20. For socially-housed mice, *r*^2^ = 0.0003, *p* = 0.95, *n* = 16.

Two-way ANOVA of FosB-positive cells for each region revealed no significant effects of CRFR1 antagonism, nor interactions between antagonism and housing (Supplementary Figure 10). Therefore, we ran analyses of correlations for FosB and average 8% alcohol intake (Figure 13) and FosB and average ethanol preference (Supplementary Figure 12) for individually- and socially-housed mice. No relationships were observed between FosB immunoreactivity and average 8% g/kg ethanol intake for individually- or socially-housed mice in the NAcc or CeA (Figure 13). However, positive correlations between average g/kg alcohol intake and FosB-positive cells were observed in the NAcs and EW for individually-housed mice (*p* <0.05 for both correlations), while no relationship between these measures were observed for socially-housed mice (Figure 13).

## Discussion

The experiments described here for the first time directly demonstrated the facilitating effects of social housing on alcohol consumption in adult male C57BL/6J mice. The use of RFID tracking in the HM2 cages allowed us to analyze drinking behavior of individual subjects within social housing with high temporal specificity. Overall, we found that socially-housed mice consumed significantly more ethanol and water compared to individually-housed mice, and that this increased consumption is driven by a combination of increased number of drinks and increased drink size within this group.

As the HM2 system allows for detailed temporal analyses, we chose to examine the patterns in intake, number of drinks, and channel entries at an hourly interval for each baseline ethanol concentration presented. We found that while significant interactions between housing condition and time were observed for g/kg ethanol and water intake when the 4% concentration was available, posthoc analyses revealed no significant differences in intake at specific timepoints. Similar findings were observed at the 6% concentration, with the addition of an interaction between housing condition and time being observed for ethanol and water drinks as well, and as with the 4% concentration, posthoc analyses revealed no significant differences between socially- and individually-housed mice at specific timepoints. However, when ethanol concentration was increased to 8% and significant interactions between housing condition and time were, again, observed for mls and g/kg ethanol and water intake and for ethanol and water drinks, posthoc analyses revealed several timepoints at which socially-housed mice consumed significantly more fluid than individually-housed subjects. Collectively, these findings suggest that the difference in intake between individually- and socially-housed mice became stronger as the ethanol concentration increased. This concept is visualized in the baseline hourly datasets, in which the peaks of ethanol consumption are not distinct until the 8% concentration. When this concentration was presented, socially-housed mice exhibit two distinct peaks in intake (at ~2 h and 10 h into the dark cycle), during which their intake was significantly higher than that of individually-housed mice during these timepoints.

It should be noted, though, that while our data suggest that social enrichment may promote, while environmental enrichment alone may attenuate fluid intake, we did not measure food intake across this study. Therefore, we cannot determine whether socially-housed mice also consumed more food than individually-housed mice, and it is possible that this consummatory behavior also differs as a function of housing condition. To assess the generalizability of these findings, future studies should be conducted to assess the effect of housing condition on food intake as well as the intake of other liquids, such as sucrose or saccharin solutions.

In addition to differences in fluid intake, we also found significant differences in the number of non-consummatory ethanol and water channel entries between socially- and individually-housed mice. Perhaps surprisingly, in contrast to intake data, these findings demonstrated that individually-housed mice made significantly more channel entries than socially-housed mice, across all ethanol concentrations presented and in both fluid channels. Although the cause of this increase is unknown, the CRFR1 antagonism-induced attenuation of this behavior, specifically within individually-housed mice, suggest that the increase in channel entries may be a manifestation of anxiety-like behavior. It has, in fact, been shown that male mice housed individually during adolescence or adulthood exhibit increased anxiety-like behavior in open field, evidenced by decreased time spent in the center of the apparatus, compared to socially-housed controls (Lander et al., 2017). This study also found that individually-housed mice had generally higher locomotor activity than socially-housed controls (Lander et al., 2017). Our previous study also observed higher levels of c-Fos in the CeA in individually vs. socially-housed mice housed in standard shoebox cages, suggesting potentially higher levels of anxiety in individually-housed mice (Robins et al., 2020). However, because CRFR1 antagonism did not decrease channel entries in socially-housed mice in our study, it is unlikely that the reduced channel entries in the individually-housed group were simply due to sedative effects of the treatment. Therefore, while it is possible that increased anxiety-like behavior in individually-housed mice may have caused increased non-consummatory channel entries, because anxiety-like behavior was not directly assessed in this study, no definitive conclusions on the cause of this behavior can be made.

As mentioned above, we found that a single, systemic treatment with the CRFR1 antagonist CP-376,395 caused a decrease in non-consummatory channel entries for individually-housed mice. In contrast, the effects of this treatment on fluid intake were consistent across housing conditions. Specifically, we observed CRFR1 antagonism-induced decreases in ethanol and water intake, demonstrating that this treatment/dose is not selective for attenuating ethanol intake. Interestingly, the effects on water were longer lasting, in that a significant effect of CRFR1 antagonism was observed in cumulative 3 and 24 h datasets, whereas the suppressive effect on ethanol intake was only observed at the 3 h timepoint. These findings are, in part, consistent with those from a previous study in our lab which assessed the effects of CP-376,395 on ethanol two-bottle choice in mice housed individually in standard, shoebox cages (Potretzke et al., 2020). More specifically, in this previous study, treatment-induced decreases in both ethanol and water intake only lasted 3 h, while in the current investigation, decreased water treatment was observed up to 24 h post-treatment. It is possible that these differences in treatment effects are related to the lower ethanol intake levels of HM2 mice when compared to those housed in standard, shoebox cages. Importantly, while CRFR1 antagonism was originally considered selective for excessive drinking observed in models of dependence and binge-like drinking (Valdez et al., 2002, Finn et al., 2007, Funk et al., 2007, Sparta et al., 2008), our findings are in agreement with previous demonstrations that this treatment is not specific for ethanol (Giardino and Ryabinin, 2013, Potretzke et al., 2020).

Twenty-four hours after CP-376,395 treatment, the first cohort of animals was allowed to consume water and 8% ethanol for 3 h before euthanasia and blood collection to measure BECs. While we observed no differences between individually- and socially-housed mice, BECs ranged from 5.97 to 77.58 mg/dL. The low BECs in the majority of animals are consistent with the fast metabolism of ethanol in mice, but it is notable that we still observed relatively high alcohol levels in two subjects. To test whether ethanol consumption in HM2 cages resulted in pharmacologically-relevant levels of ethanol across all animals, we also assessed levels of FosB, a marker of long-term, repeated neuronal activation (Nestler et al., 2001) in brain regions known to respond to ethanol exposure: NAcc, NAcs, CeA, and EW. For this reason, 24 h after CP-376,395 treatment, the subsequent 4 cohorts were allowed to consume water and 8% ethanol for 4 h before euthanasia and brain collection for FosB immunoreactivity. While no effects of treatment or social housing on FosB levels in these regions were detected, we observed positive correlations between average ethanol intake/preference and NAcs and EW FosB immunoreactivity in individually-housed mice, but not in socially-housed subjects. The positive correlation between FosB and ethanol intake is attributable to a substantial number of low-drinking subjects among individually-housed mice. This correlation indicates that higher alcohol consumption is associated with higher FosB levels in NAcs and EW. Since no low-drinking mice were present among the socially-housed mice, no correlation with FosB was observed in this group of animals, suggesting saturation of the FosB response. Thus, our FosB data are in agreement with the idea that ethanol consumption resulted in activation of cells in select brain regions, indicative of physiological effects of ethanol.

Lastly, we would like to address that the possible effects of resource competition and/or social rank in the social-housed mice were not directly assessed here, and that these factors could modulate ethanol consumption and even sensitivity to CRFR1 antagonism. Therefore, future studies will investigate the effects of these phenomena on ethanol intake and response to pharmacological intervention.

Overall, we found that socially-housed male mice consumed significantly more ethanol than individually-housed subjects when housed in an environmentally enriched cage. We also demonstrated the high temporal resolution of the HM2 system, which can aid in identifying patterns of fluid intake under baseline conditions and in response to pharmacological interventions. While intake levels were moderately low in this study, for both individually- and socially-housed subjects, significant correlations in FosB immunoreactivity and ethanol intake/preference were observed within two regions known to respond to ethanol exposure (the NAcs, EW). Collectively, these findings demonstrate the potential for the use of radiotracking technology in preclinical studies of alcohol use disorder in socially-housed rodents, and future studies will involve investigating these behaviors in female mice and other strains and rodent species.

## Data Availability Statement

The raw data supporting the conclusions of this article will be made available by the authors, without undue reservation.

## Ethics Statement

The animal study was reviewed and approved by Oregon Health & Science University Animal Care and Use Committee.

## Author Contributions

HF was involved in data analysis and composition of manuscript. MR was involved in experimental design, data collection and analysis, and construction of manuscript. MC was involved in data collection. AR was involved in experimental design, data analysis, and composition of manuscript. All authors contributed to the article and approved the submitted version.

## Conflict of Interest

The authors declare that the research was conducted in the absence of any commercial or financial relationships that could be construed as a potential conflict of interest.
