# Supplementary material for: Social Housing Leads to Increased Ethanol Intake in Male Mice Housed in Environmentally Enriched Cages
Source: Front Behav Neurosci. 2021 Jun 18;15:695409. doi: 10.3389/fnbeh.2021.695409 (PMC8253159; doi:10.3389/fnbeh.2021.695409)
Supplement: Supplementary file 1 [file Data_Sheet_1.docx]

Supplementary Material

**Figure S1**

***
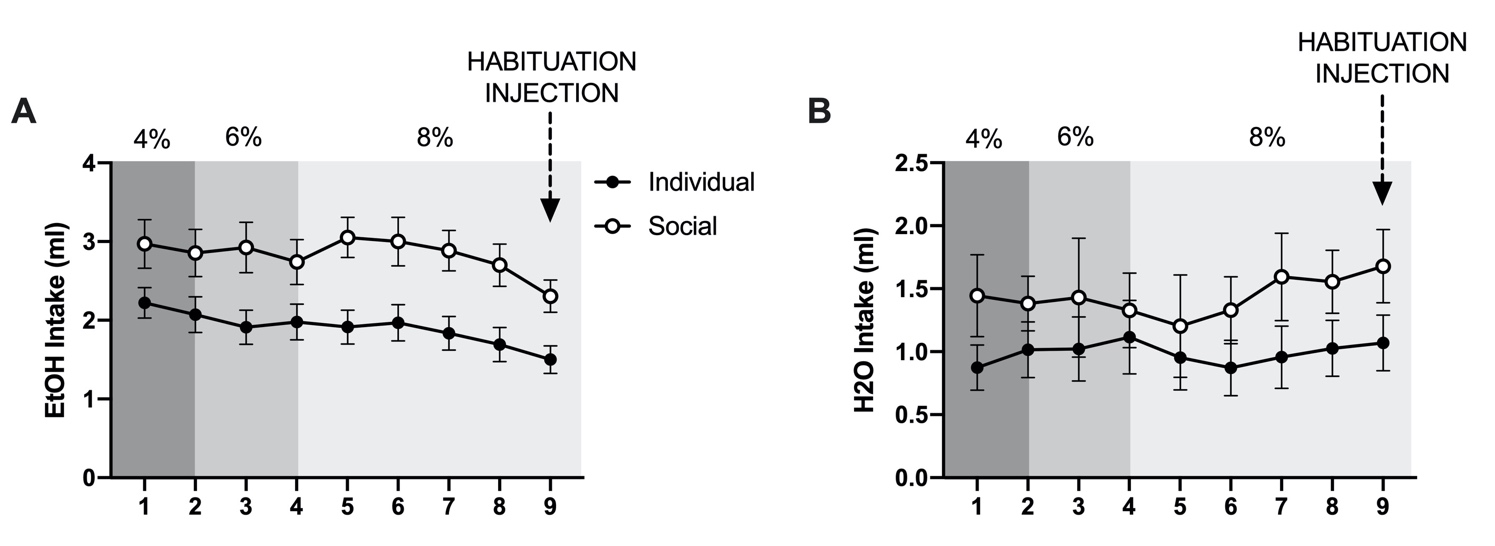
***

**Supplementary Figure 1.** **Baseline 24hr cumulative mls intake.** (A) Ethanol intake in mls. Two-way ANOVA of mls ethanol consumed revealed significant main effects of housing condition (F_1,46_=9.77, p < 0.01) and time (F_8,46_=4.11, p < 0.01) but no interaction between these factors (F_8,46_=0.55, p = 0.82). (B) Water intake in mls. Two-way ANOVA of mls water consumed revealed no effects of housing condition (F_1,46_=1.80, p = 0.19) or time (F_8,46_=0.61, p = 0.77) and no interaction between these factors (F_8,46_=0.40, p = 0.92). Data represented as mean ± SEM. n = 24/group.

**Figure S2**


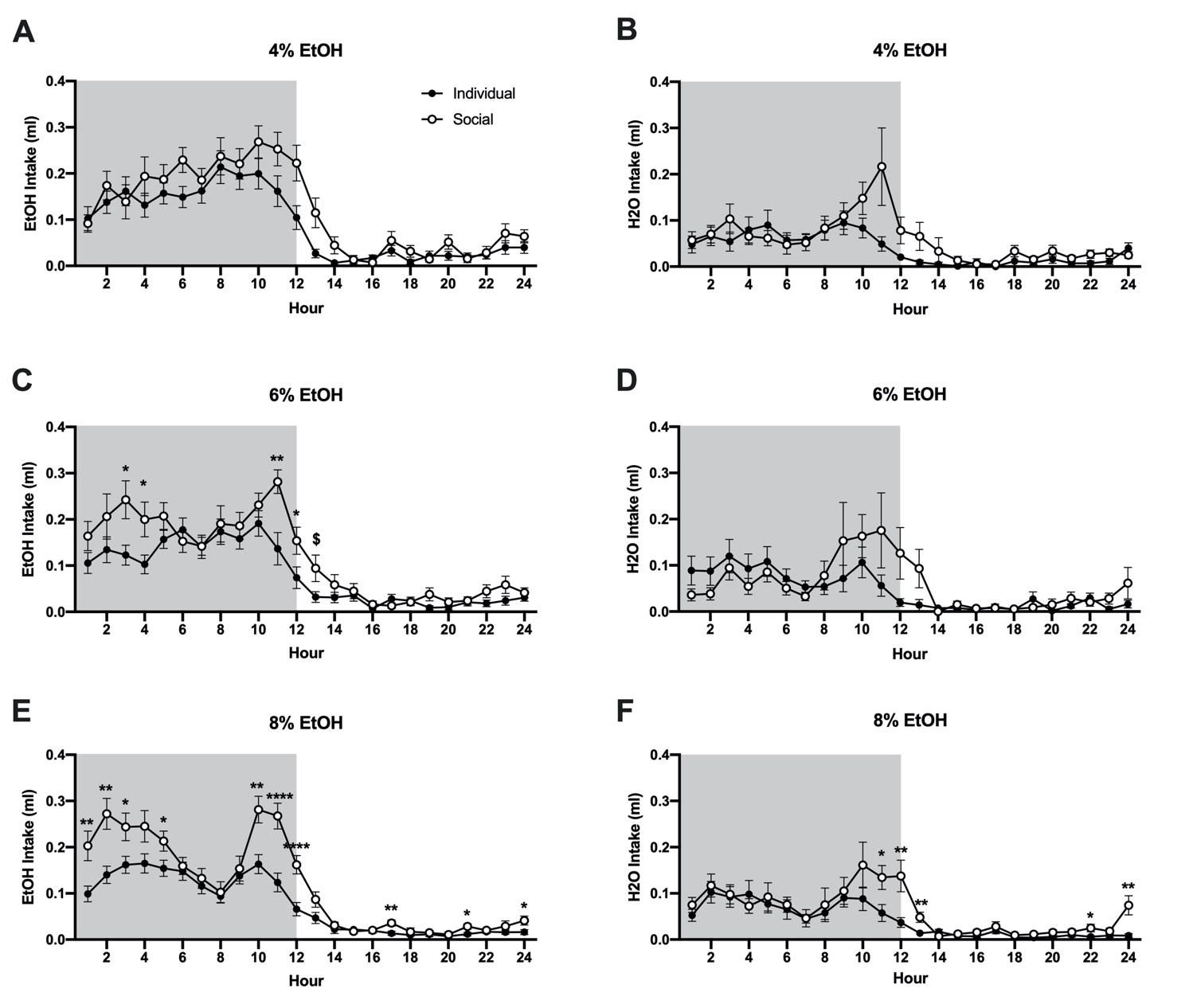


**Supplementary Figure 2.** **Baseline hourly mls intake.** Values in Fig S2 represent the mean intake across each day that each ethanol concentration was available. (A) Average hourly ethanol intake in mls at 4% concentration. Two-way ANOVA of mls ethanol intake revealed main effects of housing condition (F_1,46_=4.63, p = 0.04), and time (F_23,1058_=26.60, p < 0.0001), but no interaction between these factors (F_23,1058_=1.37, p < 0.11). (B) Average hourly water intake in mls when 4% ethanol was available. Two-way ANOVA of mls water intake revealed no effect of housing condition (F_1,46_=2.30, p = 0.14), a main effect of time (F_23,1058_=6.99, p < 0.0001), and a significant interaction between these factors (F_23,1058_=2.02, p < 0.01). However, planned comparisons analysis did not identify any differences in water intake between individually- and socially-housed mice at specific timepoints. (C) Average hourly ethanol intake in mls at 6% concentration. Two-way ANOVA of mls ethanol intake revealed main effects of housing condition (F_1,46_=6.11, p < 0.05) and time (F_23,1058_=26,49, p < 0.0001), as well as a significant interaction between these factors (F_23,1058_=2.18, p < 0.01). Planned comparisons analysis revealed significant differences in mls ethanol intake between socially- and individually-housed mice at hours 3-4 and 11-13. (D) Average hourly mls water intake in mls when the 6% ethanol concentration was available. Two-way ANOVA of mls water intake revealed no effect of housing condition (F_1,46_=0.45, p = 0.51), a main effect of time (F_23,1058_=5.79, p < 0.0001), and a significant interaction between these factors (F_23,1058_=1.96, p < 0.01). However, planned comparisons analysis did not identify any differences in intake between individually- and socially- housed mice at specific timepoints. (E) Average hourly mls ethanol intake at the 8% concentration. Two-way ANOVA revealed main effects of housing condition (F_1,46_=10.52, p < 0.01) and time (F_23,1058_=55.96, p < 0.0001), and a significant interaction between these factors. Planned comparisons analysis revealed that socially-housed mice consumed significantly more mls ethanol than individually-housed mice at hours 1-3, 5, 10-12, 17, 21, and 24. (F) Average hourly mls water intake when the 8% ethanol concentration was available. Two-way ANOVA revealed no effect of housing condition (F_1,46_=2.06, p = 0.16), a main effect of time (F_23,1058_=13.47, p < 0.0001), and a significant interaction between these factors (F_23,1058_=1.91, p < 0.01). Planned comparisons analyses demonstrated that socially-housed mice consumed significantly more mls water than individually-housed mice at hours 11-13, 22, and 24. $p = 0.05, *p < 0.05, **p < 0.01, compared to opposite housing condition. Data represented as mean ± SEM. n = 24/group. Shaded regions represent the dark cycle.

**Supplementary Figure 3.** **Baseline hourly preference and drink size.** (A) 4% ethanol preference. (B) 4% ethanol drink size. (C) Water drink size when 4% ethanol was available. (D) 6% ethanol preference. (E) 6% ethanol drink size. (F) Water drink size when 6% ethanol was available. (G) 8% ethanol preference. (H) 8% ethanol drink size. (C) Water drink size when 8% ethanol was available. Data are not able to be analyzed due to the large number of missing data points. Data represented as mean ± SEM. n = 24/group. Shaded regions represent the dark cycle.

**Figure S3**

**Figure S3**

**
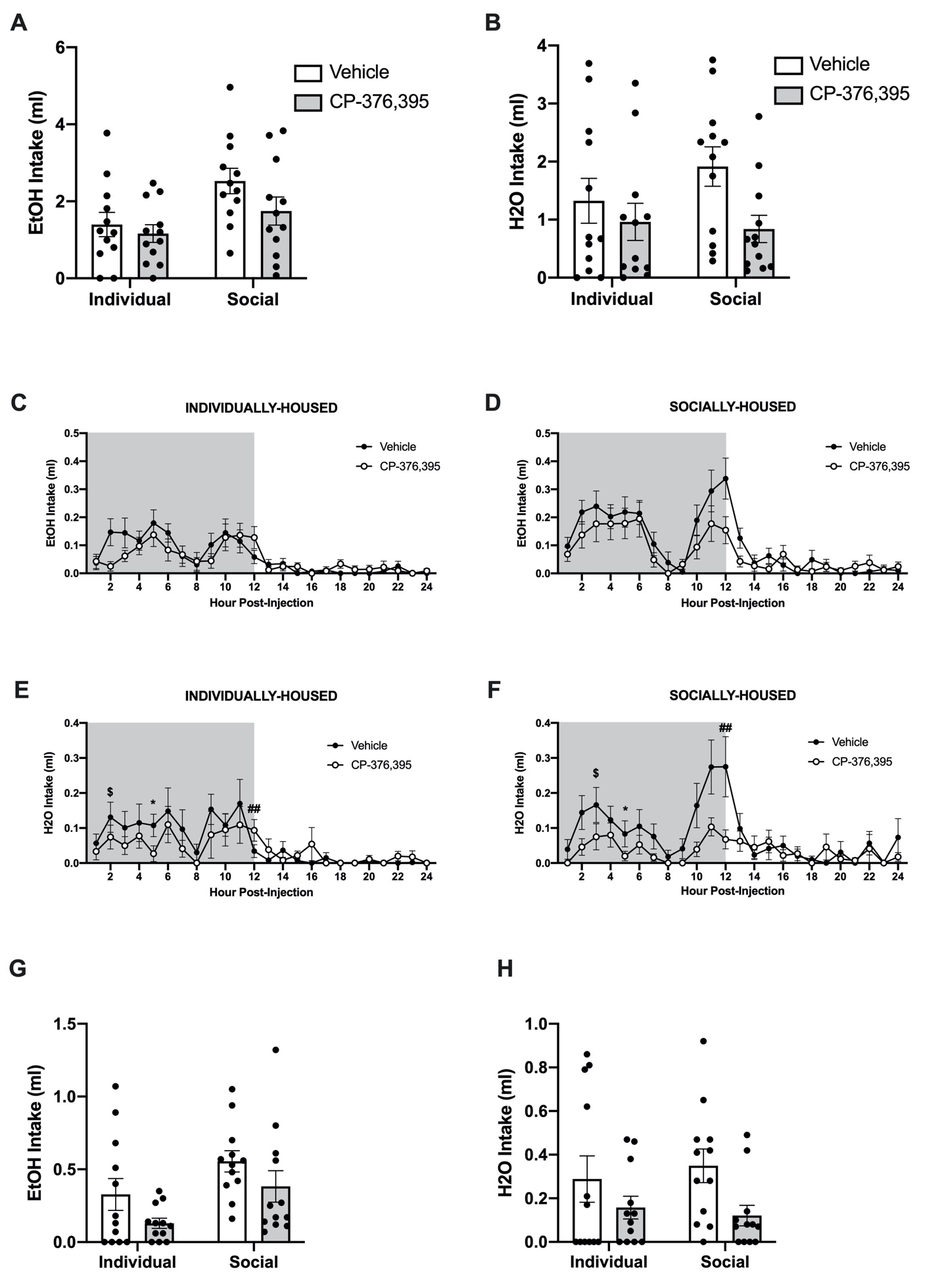
**

**Figure S4**

**Supplementary Figure 4.** **Ethanol and water intake in mls post-CP-376,395 treatment.** (A) Cumulative 24hr data for mls ethanol consumed. Two-way ANOVA of mls ethanol revealed a main effect of housing condition (F_1,44_=7.50, p < 0.01), no effect of CRFR1 antagonism (F_1,44_= 2.61, p = 0.11), and no interaction between these factors (F_1,44_=0.76, p = 0.39). (B) Cumulative 24hr data for mls water consumed. Two-way ANOVA of mls water intake revealed no effect of housing condition (F_1,44_=0.52, p = 0.48), a main effect of CRFR1 antagonism (F_1,44_=4.88, p < 0.05), and no interaction between these factors (F_1,44_=1.19, p = 0.28). (C) and (D) represent hourly mls ethanol intake and (E) and (F) hourly mls water intake for each housing condition. Data are presented as separated by housing condition for display purposes only. Three-way ANOVA of mls ethanol intake revealed main effects of housing condition (F_1,44_=7.50, p < 0.01) and time (F_23,1012_=18.62), no effect of CRFR1 antagonism (F_1,44_=2.61, p = 0.11), an interaction between time and housing condition (F_23,1012_=2.57, p < 0.0001), but no interactions between housing condition and CRFR1 antagonism (F_1,44_=0.76, p = 0.39), time and CRFR1 antagonism (F_23,1012_=1.06, p = 0.39), or all three of these factors (F_23,1012_=1.38, p = 0.11). In contrast, three-way ANOVA of mls water intake revealed no effect of housing condition (F_1,44_=0.52, p = 0.48) but significant main effects of time (F_23,1012_=8.78, p < 0.0001) and CRFR1 antagonism (F_1,44_=4.88, p < 0.05), significant interactions between housing condition and time (F_23,1012_=1.78, p < 0.05) and between CRFR1 antagonism and time (F_23,1012_=1.56, p < 0.05), but no interactions between housing condition and CRFR1 antagonism (F_1,44_=1.19, p = 0.28) or all three factors (F_23,1012_=1.24, p = 0.20). Planned comparisons analysis of mls water intake revealed a trend-level effect of CRFR1 antagonism 2hr post-treatment (p = 0.05) and a main effect of antagonism 5hr post-treatment (p < 0.05) across both housing conditions. At 9hr post-treatment, a main effect of housing condition was observed (p < 0.05), with individually-housed mice consuming significantly more mls water than socially-housed mice, with no effect of CRFR1 antagonism. Lastly, at 12hr post-treatment, a main effect of housing condition (p < 0.05) and a significant interaction between housing condition and CRFR1 antagonism (p < 0.01) were observed, with antagonist-treated subjects consuming more mls water than controls for individually-housed mice and less mls water for socially-housed mice. (E) Cumulative 3hr data for mls ethanol intake. Two-way ANOVA revealed main effects of housing condition (F_1,44_=7.61, p < 0.01) and CRFR1 antagonism (F_1,44_=4.52, p < 0.05), but no interaction between these factors (F_1,44_=0.02, p = 0.88). (F) Cumulative 3hr data for mls water intake. Two-way ANOVA revealed no effect of housing condition (F_1,44_=0.03, p = 0.87) and a main effect of CRFR1 antagonism (F_1,44_=5.85, p < 0.05), but no interaction between these factors (F_1,44_=0.43 p < 0.52). $p = 0.05, *p < 0.05, compared to vehicle-treated mice across housing condition. #p < 0.05, ##p < 0.01 represent interactions between housing condition and CRFR1 antagonism at a specific timepoint. Data represented as mean ± SEM. n=12/group. Shaded regions in C-F represent the dark cycle.

**Figure S5**

**Supplementary Figure 5. Cohort 1 ethanol data before euthanasia (first 3hr of dark cycle)**

(A) Ethanol intake in mls. (B) EtOH intake in g/kg. (C) Ethanol preference. (D) Number of ethanol drinks. (E) Ethanol drink size. (F) Ethanol channel entries. Student’s t-tests revealed no significant differences between groups for mls ethanol intake (t_1,10_=1.27, p = 0.23), g/kg ethanol intake (t_1,10_=1.16, p = 0.27), ethanol preference (t_1,10_=0.18, p = 0.86), number of ethanol drinks (t_1,10_=1.36, p = 0.20), or ethanol drink size (t_1,10_=0.18, p = 0.86). However, t-test of ethanol channel entries revealed that individually-housed mice made significantly more entries than socially-housed mice (t_1,10_=3.00, p = 0.01). Data represented as mean ± SEM. n = 4 (individually-housed), n = 8 (socially-housed). *p < 0.05.

**Figure S6**

**Supplementary Figure 6. Cohort 1 water data before euthanasia (first 3hr of dark cycle)**

(A) Water intake in mls. (B). Water intake in g/kg. (C) Number of water drinks. (D) Water drink size. (E) Water channel entries. Student’s t-test revealed no significant differences between groups for mls water intake (t_1,10_=0.77, p = 0.46), g/kg water intake (t_1,10_=0.65, p = 0.53), water drinks (t_1,10_=0.92, p = 0.38), water drink size (t_1,10_=0.13, p = 0.90), or water channel entries (t_1,10_=1.05, p = 0.32). Data represented as mean ± SEM. n = 4 (individually-housed), n = 8 (socially-housed).

**Figure S7**

**Supplementary Figure 7. Cohort 1 g/kg intake and BECs (first 3hr of dark cycle).** (A) Ethanol intake in g/kg. (B) Blood ethanol concentrations (BECs) following 3hr of intake. Student’s t-tests revealed no significant differences between groups for g/kg ethanol intake (t_1,10_=1.16, p = 0.27) or BECs (t_1,10_=0.52, p = 0.61). Data represented as mean ± SEM. n = 4 (individually-housed), n = 8 (socially-housed).

**Figure S8**

**Supplementary Figure 8. Cohorts 2-5 ethanol data before euthanasia (first 4hr of dark cycle)**

(A) Ethanol intake in mls. (B) EtOH intake in g/kg. (C) Ethanol preference. (D) Number of ethanol drinks. (E) Ethanol drink size. (F) Ethanol channel entries. Student’s t-tests revealed no significant differences between groups for mls ethanol intake (t_1,10_=1.16, p = 0.25), g/kg ethanol intake (t_1,10_=1.08, p = 0.29), ethanol preference (t_1,10_=0.89, p = 0.38), number of ethanol drinks (t_1,10_=0.31, p = 0.76), or ethanol drink size (t_1,10_=0.73, p = 0.47). However, t-test of ethanol channel entries revealed that individually-housed mice made significantly more entries than socially-housed mice (t_1,10_=2.95, p = 0.006). Data represented as mean ± SEM. n = 20 (individually-housed), n = 16 (socially-housed). **p < 0.01.

**Figure S9**

**Supplementary Figure 9. Cohorts 2-5 water data before euthanasia (first 4hr of dark cycle)**

(A) Water intake in mls. (B). Water intake in g/kg. (C) Number of water drinks. (D) Water drink size. (E) Water channel entries. Student’s t-test revealed no significant differences between groups for mls water intake (t_1,10_=0.12, p = 0.91), g/kg water intake (t_1,10_=0.06, p = 0.95), water drinks (t_1,10_=0.27, p = 0.79), or water drink size (t_1,10_=0.04, p = 0.97). However, t-test of water channel entries revealed that individually-housed mice made significantly more entries than socially-housed mice (t_1,10_=3.03, p = 0.005). Data represented as mean ± SEM. n = 20 (individually-housed), n = 16 (socially-housed). **p < 0.01.

**Figure S10**

**Supplementary Figure 10. FosB data across housing condition and previous CRFR1 antagonism treatment.** FosB immunoreactivity in (A) nucleus accumbens core, NAcc, (B) nucleus accumbens shell, NAcs, (C) central amygdala, CeA, and centrally-projecting Edinger-Westphal nucleus, EW (D). Two-way ANOVA of FosB-positive cells in the NAcc revealed no effects of housing (F_1,30_ = 0.28, p = 0.60), previous treatment (F_1,30_ = 0.03, p = 0.87), nor an interaction between these factors (F_1,30_ = 0.26, p = 0.62). Two-way ANOVA of FosB-positive cells in the NAcs revealed no effects of housing (F_1,31_ = 0.03, p = 0.87), previous treatment (F_1,31_ = 0.03, p = 0.86), nor an interaction between these factors (F_1,31_ = 1.16, p = 0.29). Two-way ANOVA of FosB-positive cells in the CeA revealed no effect of housing (F_1,29_ = 0.80, p = 0.38), previous treatment (F_1,29_ = 0.30, p = 0.59), nor an interaction between these factors (F_1,29_ = 0.72, p = 0.40). Two-way ANOVA of FosB-positive cells in the EW revealed a main effect of housing (F_1,32_=4.34, p = 0.045), but no effect of previous treatment (F_1,32_=0.36, p = 0.56), nor an interaction between these factors (F_1,32_=1.02, p = 0.32). Data represented as mean ± SEM. For NAcc, n = 20 (individually-housed), n = 15 (socially-housed). For NAcs, n = 19 (individually-housed), n = 15 (socially-housed). For CeA, n = 18 (individually-housed), n = 16 (socially-housed). For EW, n = 20 (individually-housed), n = 16 (socially-housed).

**Figure S11**


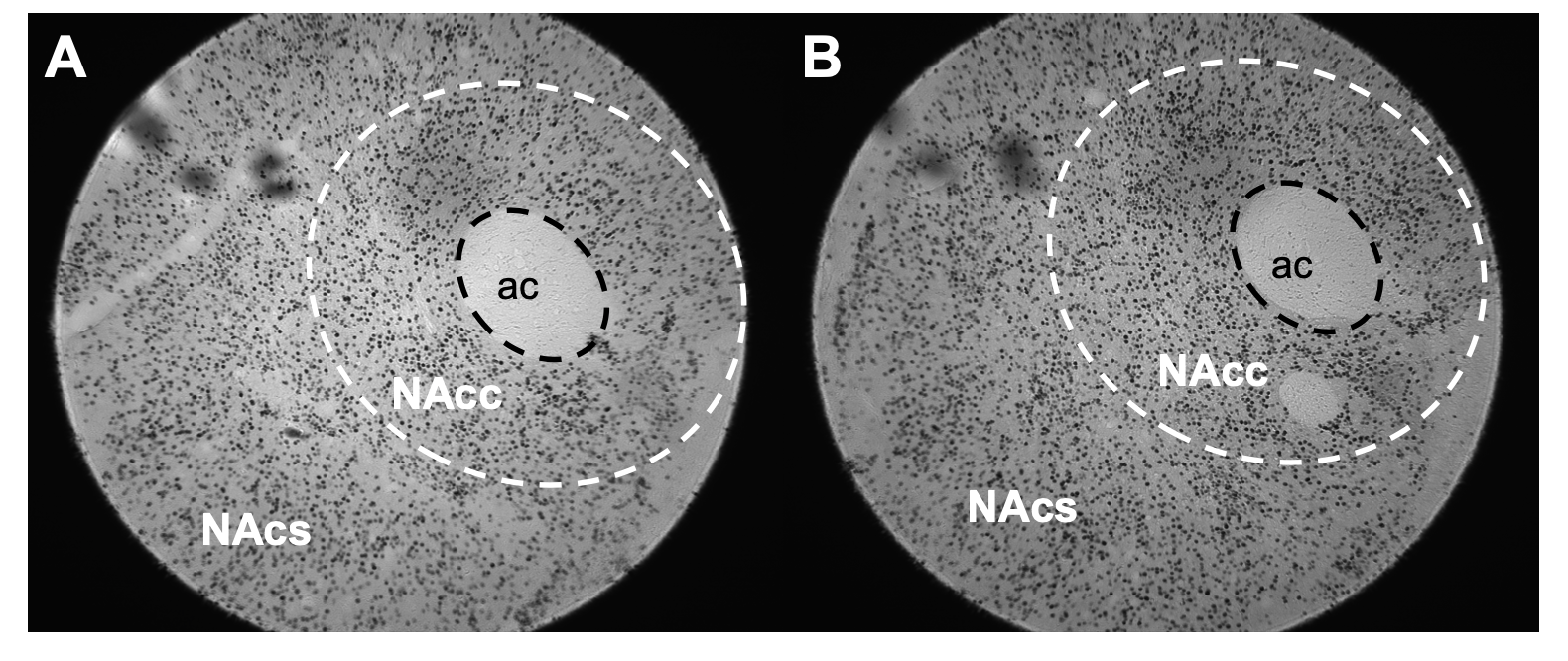

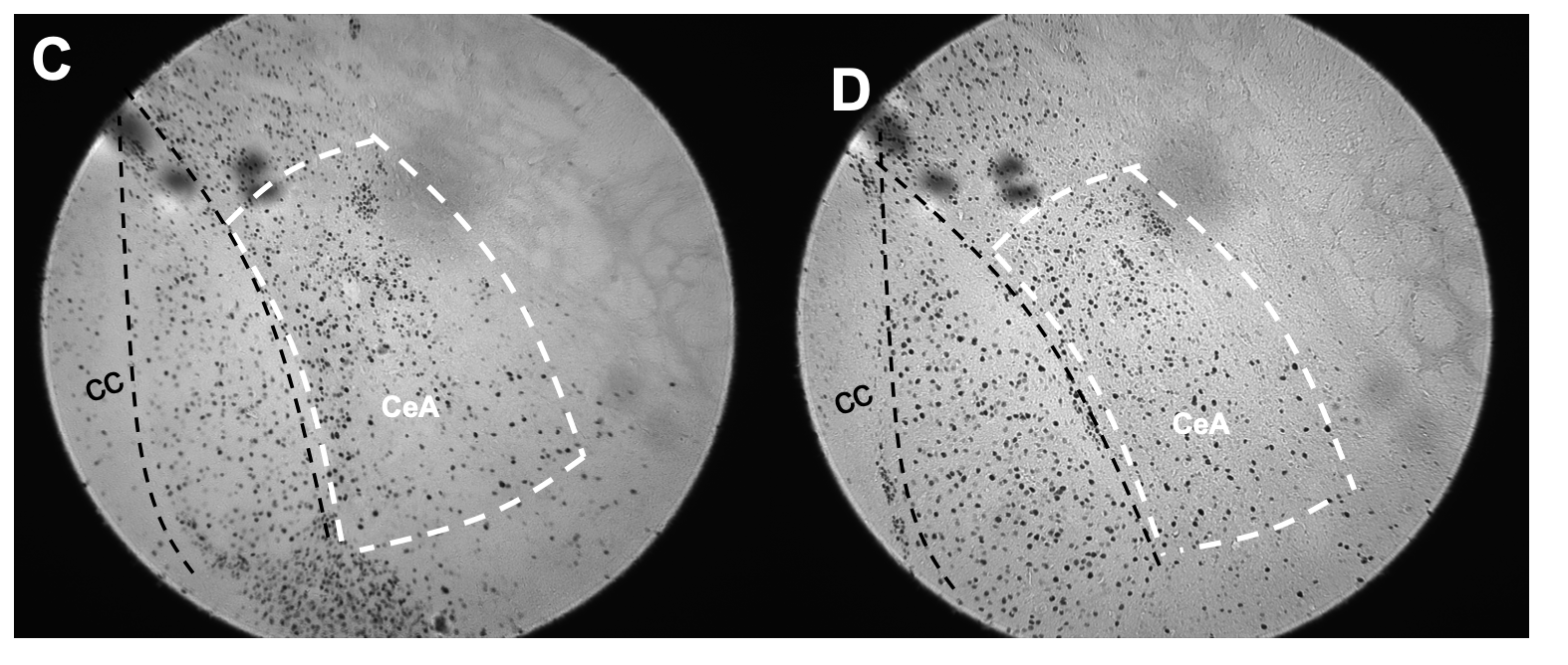

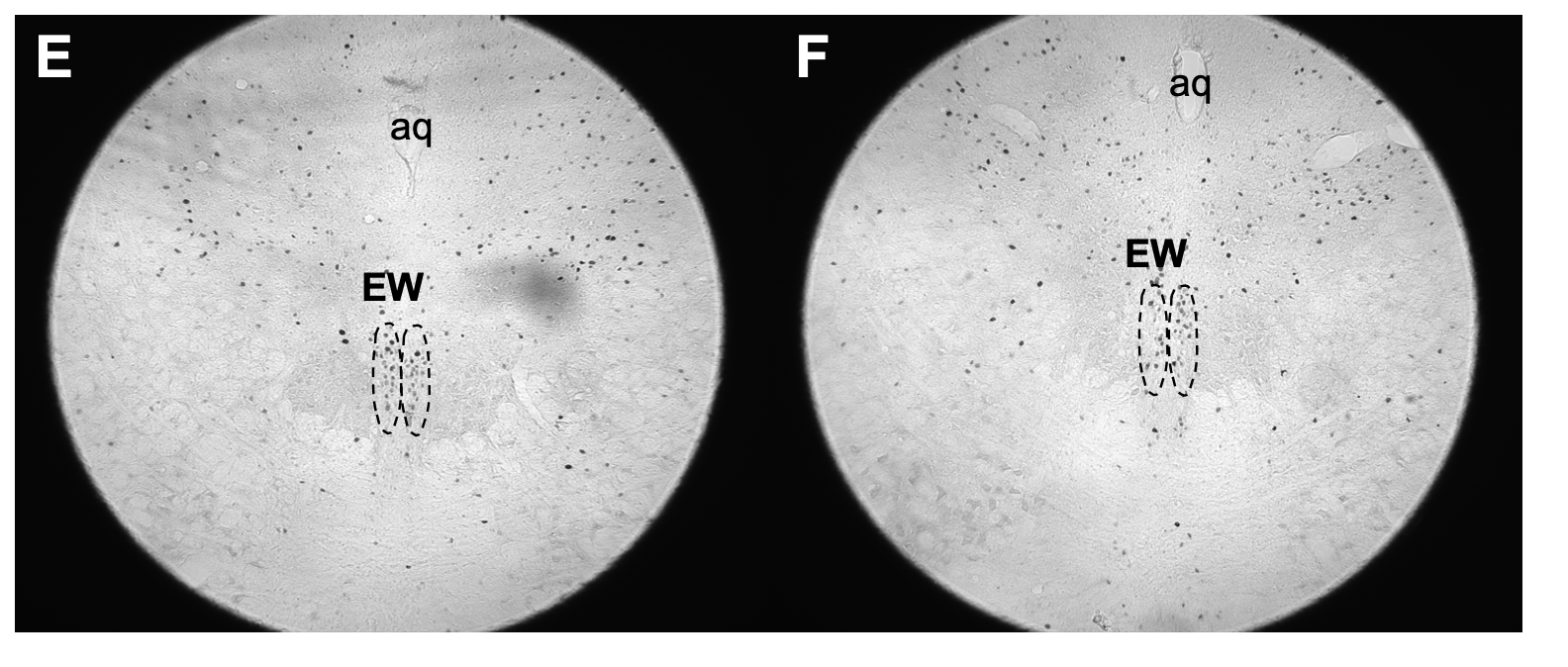


**NAcc, NAcs**

**CeA**

**EW**

**SOCIAL**

**INDIVIDUAL**

**Supplementary Figure 11. Representative FosB images.** (A) NAcc, NAcs for socially-housed subject. (B) NAcc, NAcs for individually-housed subject. (C) CeA for socially-housed subject. (D) CeA for individually-housed subject. (E) EW for socially-housed subject. (F) EW for individually-housed subject.


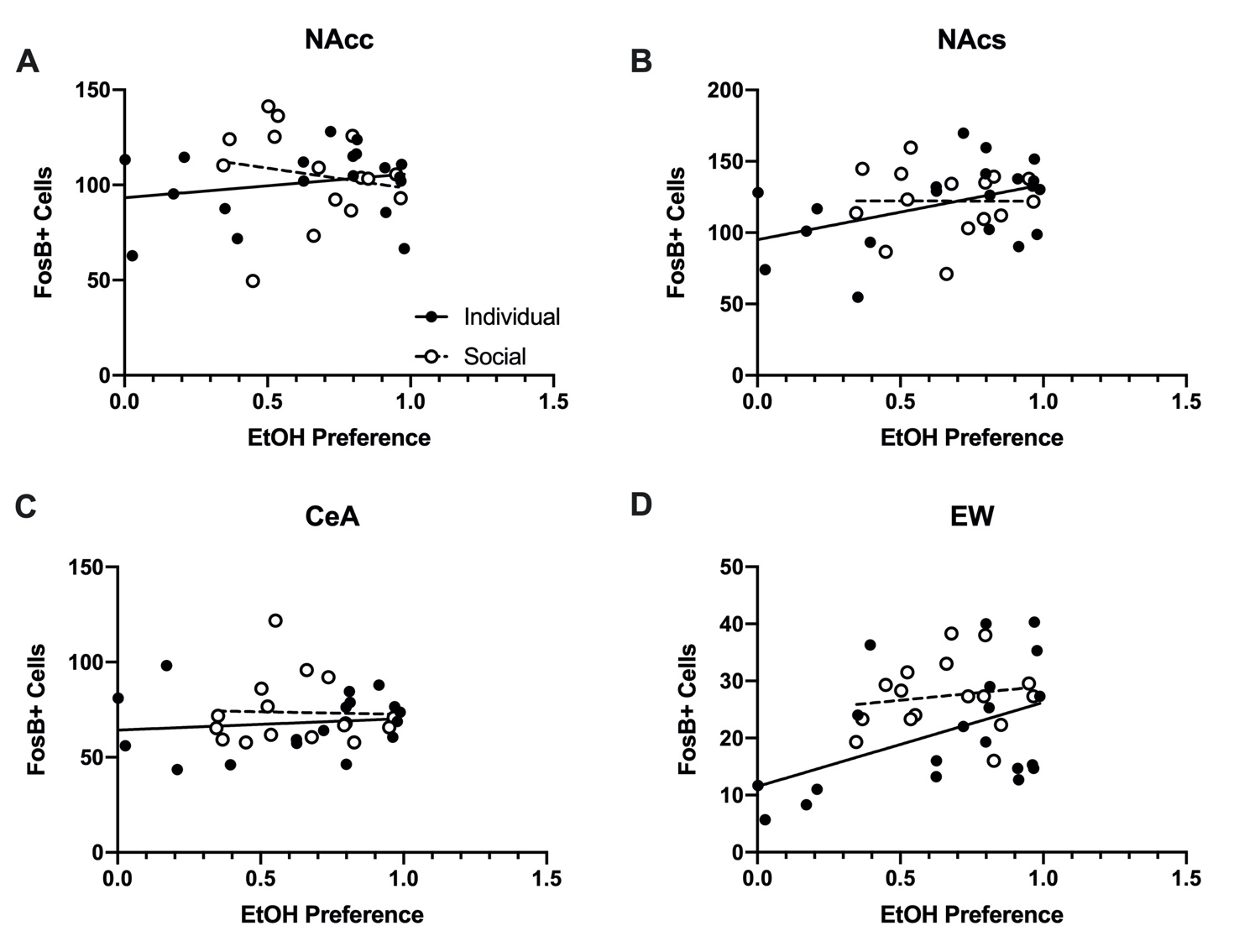


**Supplementary Figure 12. Correlations between FosB activity and average 8% ethanol preference.** Ethanol preference data represent average preference across all days of baseline 8% ethanol availability. (A) Preference correlation with FosB-positive cells in the nucleus accumbens core (NAcc). For individually-housed mice, r^2^ = 0.05, p = 0.93, n = 20. For socially-housed mice, r^2^ = 0.03, p = 0.44, n = 15. (B) Preference correlation with FosB-positive cells in the nucleus accumbens shell (NAcs). For individually-housed mice, r^2^ = 0.20, p < 0.05, n = 19. For socially-housed mice, r^2^ < 0.0001, p = 0.99, n = 15. (C) Preference correlation with FosB-positive cells in the central amygdala (CeA). For individually-housed mice, r^2^ = 0.02, p = 0.61, n = 18. (D) Preference correlation with FosB-positive cells in the centrally-projecting Edinger-Westphal nucleus (EW). For individually-housed mice, r^2^ = 0.22, p < 0.05, n = 20. For socially-housed mice, r^2^ = 0.03, p = 0.56, n = 16.

**Figure S12**
